# Nutrient availability regulates proline/alanine transporters in *Trypanosoma brucei*

**DOI:** 10.1016/j.jbc.2021.100566

**Published:** 2021-03-18

**Authors:** Alexander C. Haindrich, Viona Ernst, Arunasalam Naguleswaran, Quentin-Florian Oliveres, Isabel Roditi, Doris Rentsch

**Affiliations:** 1 Institute of Plant Sciences, University of Bern, Bern, Switzerland; 2 Institute of Cell Biology, University of Bern, Bern, Switzerland

**Keywords:** *Trypanosoma brucei*, amino acid, amino acid transport, cell metabolism, energy metabolism, gene expression, parasite metabolism, transcriptomics, transporter, BSF, bloodstream form, PCF, procyclic culture form, TCA, tricarboxylic acid, TERT, telomerase reverse transcriptase

## Abstract

*Trypanosoma brucei* is a species of unicellular parasite that can cause severe diseases in livestock and humans, including African trypanosomiasis and Chagas disease. Adaptation to diverse environments and changes in nutritional conditions is essential for *T. brucei* to establish an infection when changing hosts or during invasion of different host tissues. One such adaptation is the ability of *T. brucei* to rapidly switch its energy metabolism from glucose metabolism in the mammalian blood to proline catabolism in the insect stages and vice versa. However, the mechanisms that support the parasite's response to nutrient availability remain unclear. Using RNAseq and qRT-PCR, we investigated the response of *T. brucei* to amino acid or glucose starvation and found increased mRNA levels of several amino acid transporters, including all genes of the amino acid transporter AAT7-B subgroup. Functional characterization revealed that AAT7-B members are plasma membrane-localized in *T. brucei* and when expressed in *Saccharomyces cerevisiae* supported the uptake of proline, alanine, and cysteine, while other amino acids were poorly recognized. All AAT7-B members showed a preference for proline, which is transported with high or low affinity. RNAi-mediated AAT7-B downregulation resulted in a reduction of intracellular proline concentrations and growth arrest under low proline availability in cultured procyclic form parasites. Taken together, these results suggest a role of AAT7-B transporters in the response of *T. brucei* to proline starvation and proline catabolism.

The pathogenic trypanosomatids *Trypanosoma brucei* spp., *Trypanosoma cruzi* spp., and *Leishmania* spp. cause severe tropical diseases in humans and livestock, such as African trypanosomiasis, Chagas disease, or leishmaniasis, respectively (1). The parasitic lifestyle of trypanosomes resulted in a reduction and streamlining of their genome compared with related, free-living species of the kinetoplastids (2, 3). It favored reliance on the salvage of nutrients (4) and resulted, for example, in the development of amino acid auxotrophies (3) coupled with expansion of the respective uptake systems (2). Trypanosomes not only undergo multiple developmental transitions and linked morphological changes, but also encounter diverse environmental conditions in their insect vector and vertebrate host, to which they adapt by remodeling their metabolism (5, 6, 7, 8).

Amino acids play an important role in the metabolism and life cycle of *T. brucei* (reviewed in (9)). Proline is of particular interest, as proline biosynthesis is absent in *T. brucei*, making the parasites dependent on proline uptake for protein synthesis (8). In its insect host, the tsetse fly, the procyclic form of *T. brucei* lives in the midgut. Later developmental stages are found in the proventriculus and salivary glands (10). In these stages *T. brucei* has limited access to glucose and instead relies on the degradation of amino acids as carbon and energy sources, among which proline is the most abundant in the insect's hemolymph (11, 12).

Proline is used by *T. brucei* to fuel oxidative phosphorylation in the mitochondrion and is the main source of energy for the insect stages of the parasite (13). Proline metabolism has been studied extensively (13, 14, 15, 16, 17, 18, 19, 20) and is of fundamental importance for the survival of *T. brucei* in the tsetse fly (13). The first two enzymes of proline degradation, proline dehydrogenase and Δ^1^-pyrroline-5-carboxylate dehydrogenase (P5CDH), are essential for the parasite's grown in the absence of glucose *in vitro* (13, 14), and parasites lacking P5CDH fail to establish tsetse fly midgut infections *in vivo* (13). Downstream steps involve a partially active tricarboxylic acid (TCA) cycle and the production of pyruvate from malate. Pyruvate can be further oxidized to acetyl coenzyme A and then to acetate, or it can be used as an amino group acceptor and converted to alanine, which has to be exported from the mitochondrion and excreted from the cell (16). Alanine is used as an osmolyte in many trypanosomatids, contributing to a large portion of the cellular amino acid pool (21), and was also shown to be a main excretion product in hypo-osmotic stress responses in *Leishmania* spp. (22, 23) and *T. cruzi* (24).

Because proline degradation is linked to the TCA cycle, it is interesting that TCA cycle intermediates can initiate the differentiation from short stumpy bloodstream forms to procyclic forms (25). This differentiation can also be triggered by depriving stumpy forms of glucose, which concomitantly induces upregulation of enzymes involved in proline degradation (17). Similarly, upregulation of proline metabolism as well as an increase in proline uptake and consumption was shown in procyclic culture forms (PCF) shifted from glucose-containing to glucose-free medium (14).

In the tsetse fly, *T. brucei* has to compete for nutrients, including proline, which is used by the insect as energy source for flight muscles (11, 26). Proline availability undergoes high fluctuations during starvation periods or insect flight (11). In contrast, in the bloodstream of the mammalian host, nutrients are readily replenished, and a high availability of glucose allows the parasite to utilize this sugar as its main energy and carbon source. *T. brucei* can not only be found in the bloodstream, but also in the skin (27), adipose tissue (28) and, during the second stage of parasitemia, in the cerebrospinal fluid (29), all being rather poor in nutrients compared with blood (30). Therefore, *T. brucei* has to be able to adapt to varying nutritional conditions in the insect and the vertebrate host, both possibly involving the regulation of amino acid transporters.

While many amino acid transport activities have been determined using whole trypanosomatids (9), few individual transporters have been characterized at the molecular level in *T. brucei* (31, 32, 33). While it was shown that procyclic form *T. brucei* upregulates proline transport in the absence of glucose (14), the transporters involved are still unknown. In *Leishmania* the selective proline/alanine transporter LdAAP24 was shown to be essential to fuel the cellular proline pool and be involved in the response to hypotonic stress (23). The LdAAP24 homolog in *T. cruzi* (TcAAAP069) also transports proline (34). The most closely related transporter in *T. brucei*, TbAAT6, was identified as the entry point for eflornithine (32, 35, 36) and also supports uptake of mainly neutral amino acids, including proline (32), but its involvement in proline metabolism was not tested.

Parasites can respond to amino acid availability. *Leishmania donovani* can sense a reduction in environmental arginine and reacts by upregulating the high-affinity arginine transporter LdAAP3 in a mitogen-activated protein kinase-dependent way (37, 38). High-affinity arginine transporters have been shown to be essential for *T. brucei* PCF and blood stream form (31), but the regulation of these and other amino acid transporters by nutrient availability in *T. brucei* has not been studied so far.

Transcriptome and proteome analyses in *T. brucei* identified multiple amino acid transporters that were regulated stage specifically (25, 39, 40, 41, 42, 43, 44, 45, 46). This response may reflect the different nutritional environments and amino acid availability within the different host tissues, but also the change in energy metabolism between the mammalian and the insect life-cycle stages. We have looked for amino acid transporters whose transcripts are regulated in response to amino acid starvation, as might be encountered by trypanosomes in the skin tissue or the cerebrospinal fluid in the mammal, upon change of host or during starvation of the tsetse fly. We identified transcripts of amino acid transporters from three loci that were upregulated upon nutrient starvation. Further characterization of three of these genes belonging to the amino acid transporter 7 family (AAT7, (47)) showed that they encode high and low-affinity proline transporters that are essential in *T. brucei* procyclic form under proline limitation.

## Results

### Amino acid transporters of the *T. brucei* AAT7 locus are upregulated in response to amino acid or glucose starvation

To test whether amino acid deprivation has an impact on transcript levels of amino acid transporters, we performed transcriptome analyses of *T. brucei* under different starvation conditions. For this purpose we prepared modified culture media that allowed us to assess the response to amino acid starvation in both procyclic form and bloodstream form (BSF) *T. brucei* and the response to glucose starvation in PCF. For these experiments we used 29-13 (Lister 427 procyclic form, (48)) cultivated in commercial SDM79 (including 10% FBS) (49). To start starvation, SDM79 was replaced by different formulations of our home-made starvation medium, SDM79S, which closely resembles the formulation of SDM79, but lacks FBS (for composition see Table 1 and for details Table S1). As a control, fresh commercial SDM79 (+10% FBS) was used. Starvation media contained glucose and all amino acids (SDM79S+AA, *i.e.*, control for lack of FBS), glucose but no amino acids (SDM79S-AA), or lacked glucose, but contained all amino acids and 50 mM N-acetyl glucosamine (GlcNAc) to reduce uptake of residual glucose (SDM79S-G). A similar experimental setup was chosen for starvation of bloodstream form NY-SM cells (48). Cells were first cultured in HMI-11 (containing 10% (v/v) FBS, based on commercial IMDM) (50) and then incubated either in fresh HMI-11 or starvation medium HMIS (for composition see Tables 1 and S1) containing all amino acids but no FBS (HMIS+AA) or in HMIS containing no amino acids and no FBS (HMIS-AA). After the starvation period, RNA was extracted and submitted for RNA sequencing (RNA-seq) and mapped to the genome of *T. brucei* Lister 427_2018 (51). RNA-seq resulted in at least 20 million reads per sample and could be mapped with an efficiency of >80% to the reference genome. For comparative transcriptome analysis, reads were mapped to the annotated coding sequences of *T. brucei* Lister 427_2018, which accounted for 34–47% of all reads, *i.e.*, ≥ nine million reads per sample. Mapped reads per gene were normalized to the total number of mapped reads per sample to obtain RPM (reads per million) values but were not normalized to the length of the transcript. We could identify mapped reads of approximately 14,400 genes, from which we removed all pseudogenes genes and genes with less than 100 mapped reads within the coding region of the gene, leaving around 8200 genes for PCF data and 8600 genes for the BSF data. Data of two independent biological replicates are shown (Tables 2 and S2).

**Table 1:** Composition of media used for starvation and RNAi experiments

A	Media	SDM79+FBS	SDM79S+AA	SDM79S−AA	SDM79S−P	SDM79S−AA+P	SDM79S−G	SDM79S−G+0.1P
Basic composition	Amino acids (AA)	+	+	-	+	-	+	+
	Glucose (G)	+	+	+	+	+	-	-
	Proline w/o FBS [μM]	5344	5342	0	0	5342	5342	534
Starvation RNA-seq and/or qRT-PCR	+ FBS	10%	0%	0%	0%	0%	0%	
	Final proline conc. [μM]	5474	5342	0	0	5342	5342	
RNAi	+ FBS	10%	10%		10%		10%	10%
	Final proline conc. [μM]	5474	5472		130		5472	664


B	Media	HMI-11	HMIS+AA	HMIS−AA	HMIS−G	CMM+S
Basic composition	Amino acids (AA)	+	+	-	+	some
	Glucose (G)	+	+	+	-	+
	Proline w/o FBS [μM]	347	347	0	347	0
Starvation RNA-seq and/or qRT-PCR	+ FBS	10%	0%	0%	0%	0%
	Final proline conc. [μM]	477	347	0	347	0
RNAi	+ FBS	10%				10%
	Final proline conc. [μM]	477				130

Media composition (Comp.) used for the starvation experiments (RNA-seq and qRT-PCR) and for growth of RNAi lines of (*A*) PCF and (*B*) BSF cell cultures. Media were prepared with amino acids (AA), glucose (G), proline (P), fetal bovine serum (FBS), or without. For the RNAi experiments all media used contained 10% FBS. Detailed media compositions are described in Table S1. +, present; -, omitted.

**Table 2:** Starvation-regulated genes in *T. brucei* PCF

Selected transporters and enzymes upregulated in the absence of amino acids or glucose. Amino acid transporters of unknown and known (Tb927.8.8290, (33); Tb927.8.5450, (32); Tb927.8.4710, (31)) function, enzymes involved in proline catabolism, and enzymes of the TCA cycle not involved in proline metabolism. The table shows the fold change of mRNA levels between cells starved (2 h or 6 h) for amino acids (−AA) or glucose (−G) and nonstarved (+AA) PCF cells, for two biological replicates (I & II). The effect of FBS is shown by comparing cells grown in SDM79 + 10%FBS (FBS) with cells grown in starvation medium SDM79S including amino acids and glucose, but no FBS (+AA), *i.e.*, FBS/+AA. Red shading indicates downregulation and green shading upregulation. For each 427_2018 gene identified we matched the corresponding syntenic 927 gene, if possible, and included the gene name and product description for convenience. For amino acid transporters we included the locus-based nomenclature (47) instead of the gene name.

In amino-acid-starved PCF among the ten most highly upregulated transcripts, we found four amino acid transporters that showed more than 4-fold upregulation after 6 h starvation, compared with cells grown with amino acids (Tables 2 and S2). Three of the genes (Tb927.8.7610/7630/7640; 427_2018 does not contain a gene copy of Tb927.8.7620) belong to the amino acid transporter AAT7 locus (47). Additionally, the amino acid transporter Tb927.11.15960 (AAT17.2) showed a similar increase in expression. Messenger RNA levels of all these transporters were generally higher in medium depleted of amino acids than in medium lacking glucose (Table 2). The RNA-seq data further showed a small effect on the transcript levels of the ornithine transporter Tb927.8.8290 (AAT10.1, (33)), one additional gene of the AAT7 locus (Tb927.8.7650), and also a minor effect on the putative amino acid transporter Tb927.8.8300. Transcript levels of AAT6 (Tb927.8.5450) that was previously reported to transport neutral amino acids including proline (32), or the arginine transporter Tb927.8.4710 (AAT5.2, (31)), and other putative amino acid transporters were either not altered or not upregulated (Tables 2 and S2).

**Figure 1:**
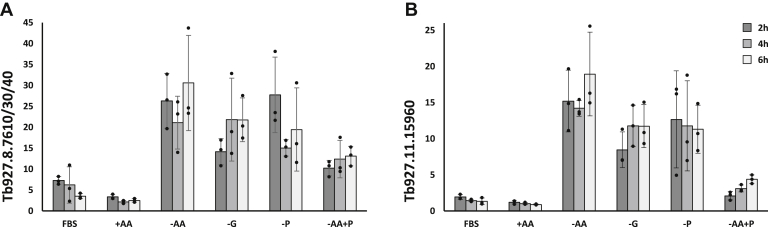
**qRT-PCR analysis of starved PCF.** qRT-PCR analysis of amino acid transporter expression of *T. brucei* 29-13 starved for 2 h (*dark gray*), 4 h (*medium gray*), or 6 h (*light gray*) of glucose (G), amino acids (AA) or proline (P). *y*-Axis shows the expression of (*A*) Tb927.8.7610/30/40 or (*B*) Tb927.11.15960s, relative to the reference gene TERT. Media composition as described in Table 1 and Table S1. FBS, commercial SDM79 + 10%FBS; +AA, starvation medium SDM79S containing amino acids and glucose, but no FBS; −AA, starvation medium SDM79S containing glucose, but no amino acids and no FBS, −G; starvation medium SDM79S containing amino acids, but no glucose and no FBS; −P, starvation medium SDM79S containing amino acids and glucose, but no proline and no FBS; −AA+P, starvation medium SDM79S containing glucose and proline, but no other amino acids. Bars show the average of three independent experiments, error bars represent SD, black dots show individual data points.

To validate the RNA-seq results, expression of selected amino acid transporters, which were upregulated (Tb927.8.7610/30/40, Tb927.11.15960), slightly altered (Tb927.8.8290, Tb927.8.8300), or not affected (Tb927.8.4710 and Tb927.8.5450) by starvation, was verified in an independent experiment by qRT-PCR using telomerase reverse transcriptase (TERT, Tb927.11.10190) as a reference gene. By using a single primer for the three genes Tb927.8.7610/30/40, we further could confirm higher transcript levels when cells were depleted of either amino acids or glucose, compared with control conditions where both compounds were present. To better describe time dependence of transcript upregulation, three time points (2 h, 4 h, and 6 h), were used for the qRT-PCR analyses. While a time dependence of Tb927.8.7610/30/40 mRNA levels was observed in the RNA-seq experiments, this could not be seen in the experiments analyzed by qRT-PCR (Fig. 1*A*). We further confirmed the high upregulation of the putative amino acid transporter Tb927.11.15960 upon deprivation of amino acids or glucose (Fig. 1*B*). The ornithine transporter Tb927.8.8290 showed a less pronounced upregulation under amino acid starvation in the qRT-PCR analysis (Fig. S1*A*), but still a general trend for upregulation in starved compared with nonstarved cells (Table 2). Similarly, the changes during deprivation of amino acids and glucose starvation observed for Tb927.8.8300 were not as pronounced using qRT-PCR compared with RNA-seq and were mostly seen during later time points of starvation (6 h). Moreover, we also observed reduced Tb927.8.8300 transcript levels in medium lacking FBS (Fig. S1*B*). The other transporters analyzed by qRT-PCR *i.e.*, the arginine transporters of the AAT5 locus (Tb927.8.4710, Tb927.8.4720, Tb927.8.4730, Tb927.8.4740, and Tb927.8.4750, (31)) were slightly upregulated upon glucose depletion, although the measurements showed strong variability (Fig. S1*C*), while the neutral amino acid transporter AAT6 (Tb927.8.5450) (32) showed no significant changes in expression upon deprivation of glucose or amino acids (Fig. S1*D*). The lack of FBS in all formulations of the starvation media caused only minor changes to amino acid transporter expression within the timeframe of the experiment (Table 2, Fig. 1 and Table S2).

Having seen that the transcripts of the AAT7-B transporter (Tb927.8.7610/30/40) become upregulated during amino acid starvation, we tested if the upregulation could be attributed specifically to starvation for a single amino acid. Of particular interest was proline due to its role as energy source in the insect stages of *T. brucei*. We tested if upregulation was due to the need for proline or if other amino acids had the same effect. Analysis of RNA extracted from cells starved for proline (SDM79S-P) showed increased expression of the Tb927.8.7610/30/40 transcripts, but we also observed upregulation if cells were starved of all other amino acids except proline (SDM79S-AA+P, Fig. 1*A*). Transcript levels of Tb927.11.15960, which were highly upregulated upon deprivation of amino acids or glucose, were also high when only proline was omitted form the medium. When all other amino acids were removed, but proline was present, Tb927.11.15960 induction was much lower, indicating a selective response to proline (Fig. 1*B*). We also tested other amino acid transporters for a selective response to proline deprivation, but found no significant response (Fig. S1).

**Figure 2:**
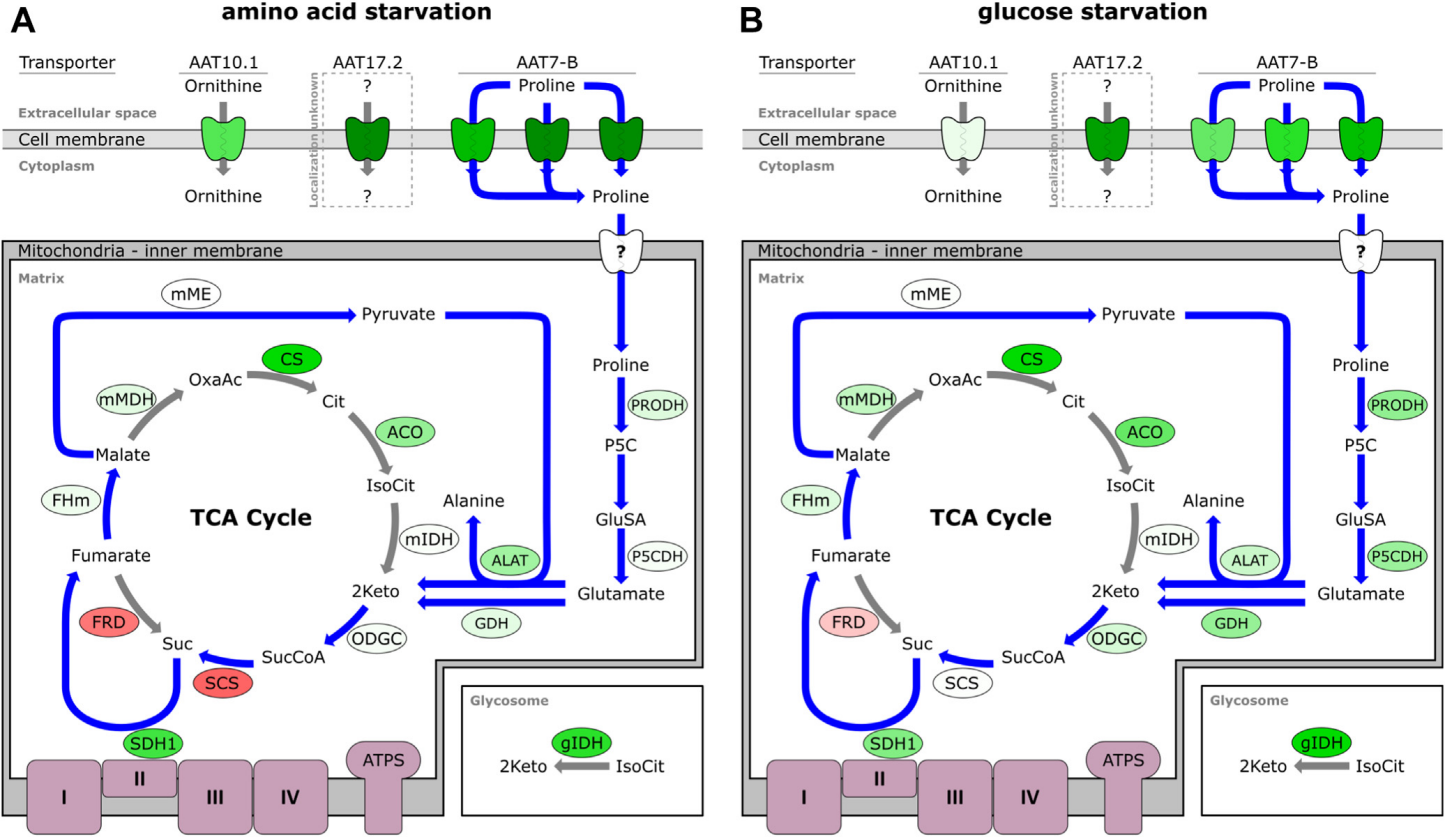
**Overview of metabolic genes regulated in starved *T. brucei* PCF.** The scheme shows enzymes and transporters regulated in PCF cells starved of (*A*) amino acids or (*B*) glucose. *Blue arrows* show pathways involved in proline metabolism. Enzymes (in *bubbles*) and amino acid transporters (AATs, *apple shape*) are color coded according to Table 2. Color is intermediate of replicates I and II after 6 h of starvation. Transporter: AAT10.1, ornithine transporter Tb927.8.8290; AAT17.2, uncharacterized transporter Tb927.11.15960, AAT7-B, proline transporter Tb927.8.7610, Tb927.8.7630, Tb927.8.7640 (from left to right). ACO, aconitase; ALAT, alanine aminotransferase; Cit, citrate; CS, citrate synthase; FHm, mitochondrial fumarate hydratase; FRD, NADH-dependent fumarate reductase; GDH, glutamate dehydrogenase; gIDH, glycosomal isocitrate dehydrogenase; GluSA, glutamate-5-semialdehyde; 2Keto, 2-ketoglutarate; IsoCit, isocitrate; mIDH, mitochondrial isocitrate dehydrogenase; mMDH, mitochondrial malate dehydrogenase; mME, mitochondrial malic enzyme; ODGC, 2-oxoglutarate dehydrogenase complex; OxaAc, oxaloacetate; P5C, pyrroline-5-carboxylate; P5CDH, pyrroline-5-carboxylate dehydrogenase; PRODH, proline dehydrogenase; SCS, succinyl-coenzyme A synthetase; Suc, succinate; SucCoA, succinyl-coenzyme A; SDH1, succinate dehydrogenase.

Proline is the major energy source of *T. brucei* in the tsetse fly, and cultured procyclic forms may switch to proline degradation coupled to oxidative phosphorylation upon depletion of glucose (14). We therefore also looked at enzymes involved in proline catabolism and the TCA cycle (Tables 2 and S2) and summarized the results visually (Fig. 2). The first three enzymes involved in proline degradation, *i.e.*, mitochondrial proline dehydrogenase, proline degradation, proline dehydrogenase (Tb927.7.210), Δ1-pyrroline-5-carboxylate dehydrogenase, P5CDH (Tb927.10.3210), and glutamate dehydrogenase, GDH (Tb927.9.5900), showed slightly higher expression when cells were depleted of glucose. We also saw upregulation of alanine amino transferase, ALAT (Tb927.1.3950), members of the 2-oxoglutarate dehydrogenase complex, ODGC (*e.g.*, OGDH1A, Tb927.11.1450), and members of the succinate dehydrogenase complex, SDHC (*e.g.*, SDH1, Tb927.8.6580) during starvation. Although we saw upregulation of ODGC and SDH, succinyl-CoA-synthetase, SCS (*e.g.*, SCSα, Tb927.3.2230), was downregulated upon amino acid starvation. Two other steps of proline metabolism catalyzed by mitochondrial fumarate hydratase (FHm, Tb927.11.5050) and mitochondrial malic enzyme (mME, Tb927.11.5450) did not show regulation at the mRNA level (Tables 2 and S2, Fig. 2).

Interestingly, two additional proteins of the TCA cycle, citrate synthase, CS (Tb927.10.13430) and aconitase, ACO (Tb927.10.14000), also showed higher expression during glucose and amino acid starvation. In addition, a strong upregulation of glycosomal isocitrate hydrogenase (gIDH, Tb927.11.900) mRNA was detected. While most of the enzymes for proline metabolism and the TCA cycle upregulated most strongly during glucose starvation, succinate dehydrogenase, citrate synthase, aconitase, and glycosomal isocitrate dehydrogenase also showed strong upregulation during amino acid starvation (Tables 2 and S2, Fig. 2).

We also looked at the effect of amino starvation on bloodstream form in a similar setup, but the RNA-seq data were very variable between experiments and therefore inconclusive (Table S2). The effect of amino acid starvation on BSF was also tested by qRT-PCR analysis for the same amino acid transporters as for the procyclic form. In addition, the effect of glucose starvation on BSF cells was assessed using starvation medium containing all amino acids but no glucose (HMIS-G). Both glucose starvation and amino acid starvation resulted in a reduction of Tb927.8.8290 and AAT6 mRNAs (Fig. S2, *C* and *F*). For Tb927.8.7610/30/40, Tb927.8.11.15960, and Tb927.8.8300, we could also observe a reduction in mRNA upon prolonged amino acid starvation (Fig. S2, *B* and *D*). Tb927.8.4710-50 (AAT5) transcripts analyzed by qRT-PCR showed no changes during starvation (Fig. S2).

### The starvation-induced amino acid transporter genes of the AAT7 locus belong to the same clade

**Figure 3:**
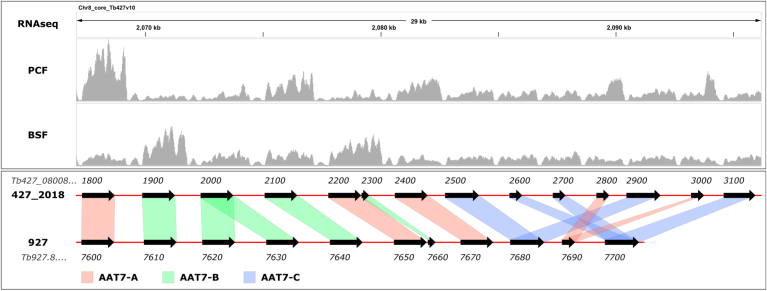
**Genes of the AAT7 locus are differentially expressed in PCF and BSF**. *Upper panel* shows the mapping coverage from an RNAseq experiments for PCF cells grown for 6 h in SDM79 + 10% FBS or BSF cells grown in HMI-11. *Lower panel* shows the architecture of the AAT7 locus using the position of the CDS for *T. brucei* Lister 427_2018 (51) or *T. brucei* TREU 927, with connection of syntenic genes. AAT7 subgroup are color coded with red for AAT7-A, green for AAT7-B and blue for AAT7-C. Genes are abbreviated with the last four digits of their TriTrypDB GeneID.

Of the genes strongly upregulated during starvation, we further characterized the transporters belonging to the AAT7 family. In *T. brucei* TREU 927 the AAT7 locus contains 11 putative amino acid transporter genes, of which two are pseudogenes (*i.e.*, Tb927.8.7660 and Tb927.8.7690). The remaining nine transporters can be phylogenetically divided into three subgroups, AAT7-A comprising Tb927.8.7600, Tb927.8.7650, and Tb927.8.7670; AAT7-B comprising Tb927.8.7610, Tb927.8.7620, Tb927.8.7630, and Tb927.8.7640; and AAT7-C, which is phylogenetically more distantly related and comprises Tb927.8.7680 and Tb927.8.7700 (Fig. 3, (47)). The genes of *T. brucei* TREU 927 are syntenic with genes in the previous database version of the genome of *T. brucei.* Lister 427 strain (Lister 427_2010, sequences obtained from TriTrypDB, version 46 (52)). The recent *de nozvo* assembly of the *T. brucei.* Lister 427 genome (referred to as Lister 427_2018) using PacBio single molecule sequencing revealed a novel architecture of the AAT7 locus, including more pseudogenes, but only three genes for the AAT7-B group (Fig. 3, (51)). During the amplification of the AAT7-B transporter genes, we found multiple SNPs between the genes amplified from genomic DNA of our *T. brucei.* Lister 427 strain and the sequences listed in the TriTrypDB database. A summary of all nucleotide and amino acid changes between the amplified sequences of our lab strain, sequences used in this study, and the database sequences (TREU 927, Lister 427_2010, Lister 427_2018; TriTrypDB, version 46 (52)) can be found in Table S3. Work using the cloned syntenic 427 genes for Tb927.8.7610, Tb927.8.7620, Tb927.8.7630, and Tb927.8.7640 will be designated as 7610, 7620, 7630, and 7640, respectively, whenever used.

Phylogenetically the AAT7 loci A and B group together with the *T. brucei* AAT4, AAT10, and AAT2 loci. These loci encode 23 amino acid transporters, which have no close homologs in *Leishmania major* and which branch with a single amino acid transporter of *T. cruzi* (2). The AAT7 locus itself might have undergone concerted evolution within *T. b. brucei*, *T. b. gambiense*, and *Trypanosoma congolense*. While *T. b. gambiense* still has homologs within the AAT7 subgroups A, B, and C, the AAT7 transporters of *T. congolense* form a group of 11 transporters separate from the TbAAT7 subgroups A and B and another separate group of three transporters to TbAAT7-C (47).

Tb927.8.7610, Tb927.8.7620, Tb927.8.7630, and Tb927.8.7640 transport proline, alanine, and cysteine when expressed in *Saccharomyces cerevisiae*

**Figure 4:**
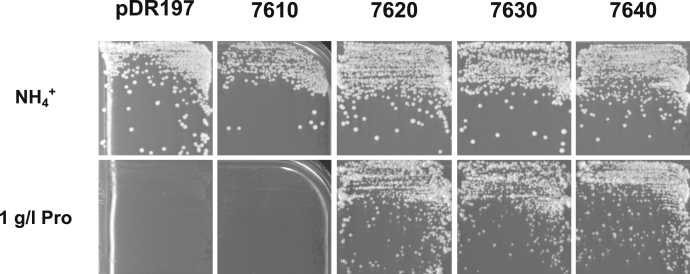
**AAT7-B transporters mediate uptake of proline.** Expression of the genes coding for 7610, 7620, 7630, and 7640 in *S. cerevisiae* mutant 22574d and growth on M.am medium containing 1 g L^−1^ proline (Pro) for 4 days and on nonselective minimal medium containing 5 g L^−1^ ammonium sulfate (NH_4_^+^) for 3 days. As control mutant 22574d transformed with the expression vector (pDR197) is shown. The overexpressed ORFs of the AAT7-B group correspond to the sequences described in Table S3.

Expression in different amino acid transport mutants of *S. cerevisiae* and growth under selective conditions revealed that 7620, 7630, and 7640 supported growth on proline and cysteine while growth on valine was very poor. (Figs. 4 and S3). 7610 complemented growth on cysteine, but showed no growth on proline (Figs. 4 and S3). None of these transporters supported growth on any of the other amino acids tested (see Experimental proocedures and Fig. S3). In contrast, Tb927.11.15960 (AAT17.2) was not able to support growth on any substrate tested so far (not shown).

**Figure 5:**
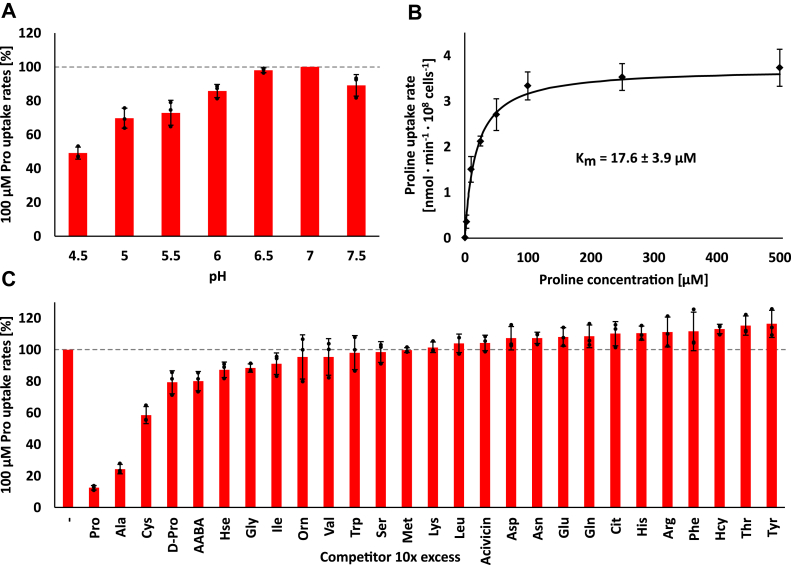
**7610 is a high-affinity proline and alanine transporter.** Uptake rates of L-[^3^H]-proline were determined using *S. cerevisiae* mutant 22574d expressing 7610. *A*, uptake rates of 100 μM proline at different pH values. Uptake rates are shown relative to the uptake rate at pH 7 (mean ± SD, n = 3). *B*, proline transport kinetics. Datapoints are means of at least three independent experiments (mean ± SD, n ≥ 3). Michaelis–Menten constant is the average of *K*_m_ values of independent experiments (mean ± SD, n = 4). *C*, uptake of 100 μM proline in the presence of different amino acids and amino acids analogs at a 10× excess (1 mM). Uptake rates are shown relative to the uncompeted proline uptake rates (−) set to 100% (mean ± SD, n = 3; 100% correspond to 6.4–7.5 nmol min^−1^ 10^8^ cells^−1^). Common L-amino acids are abbreviated with their three-letter code, other substances are D-proline (D-Pro), α-amino butyric acid (AABA), homoserine (Hse), ornithine (Orn), citrulline (Cit), and homocysteine (Hcy). Bars show the average of three independent experiments, error bars represent SD, *black dots* show individual data points.

To characterize transport properties in more detail, we measured uptake of radiolabeled cysteine in *S. cerevisiae* mutant Y01543. Because this strain only lacks the high-affinity cysteine uptake system, cysteine uptake at higher concentrations is rather high, preventing accurate determination of transport rates of the *T. brucei* transporters (not shown). Therefore, transport of proline in *S. cerevisiae* mutant 22574d expressing the ORF of 7610, 7620, 7630, or 7640 was tested. 7620, 7630, and 7640 mediated linear time-dependent proline uptake over the time course of 5 min (Fig. S4). However, for *S. cerevisiae* cells expressing 7610, we observed a decline in proline uptake (Fig. S4), and therefore measurements for this transporter were reduced to shorter times (up to 1.25 min), within which uptake of proline was linear. Transport assays at pH values between pH 4.5 and pH 7.5 showed that 7610 is functional over the entire pH range with a decrease in uptake rates at acidic pH compared with neutral pH (Fig. 5*A*). 7640 showed a stronger reduction in uptake toward acidic pH, which made measurements at low pH unreliable (Fig. S5). Both transporters exhibited maximal proline uptake rates around pH 7.0 (Figs. 5*A* and S5), which was used for all further transport assays.

By measuring the initial uptake rates at different concentrations of proline, we found a *K*_m_ of 17.6 ± 3.9 μM (mean ± SD, n = 4) for 7610 (Fig. 5*B*). Similar experiments conducted to estimate the affinity of 7620, 7630, or 7640 indicated substantially higher *K*_m_ values of 600 μM, 1000 μM, and 700 μM, respectively (Fig. S6).

To identify other potential substrates, competition experiments were performed by measuring uptake of radiolabeled proline in the presence of an excess of all proteinogenic amino acids and some amino acid analogs. For 7610, only alanine and cysteine showed strong inhibition of proline uptake, but we also observed slight inhibition with D-proline, α-amino butyric acid, homoserine, and glycine (Fig. 5*C*). The inhibition by cysteine is supported by the ability to complement growth of a *S. cerevisiae* mutant deficient in cysteine uptake (Fig. S3); while alanine was not tested in growth assays. An independent experiment in which we measured uptake of radiolabeled alanine by *S. cerevisiae* mutant YDR544 expressing 7610 confirmed that alanine enters the yeast cells (Fig. S7). The *K*_m_ of alanine estimated from the competition for proline uptake (∼48 μM) is comparable to the affinity determined in alanine transport assays (∼58 μM). Competition experiments, using *S. cerevisiae* expressing 7620, 7630, and 7640, indicated a comparable substrate specificity, with proline being the best substrate, followed by alanine and cysteine, while the ability of homocysteine to compete for proline uptake was more variable (Fig. S8, *A–C*, n = 1).

### 7610 and 7640 localize to the plasma membrane

Proline is required in the cytosol and mitochondrion for protein synthesis. In procyclic forms, mitochondrial proline is also important for energy metabolism. *In-silico* analysis showed no predicted targeting sequence for mitochondria or other signal peptides (MitoProt (53), MitoFates (54), TargetP 2.0 (55), SignalP 5.0 (56)) for any protein of the AAT7-B locus, while subcellular localization predictions suggested localization at the plasma membrane (57). The proteins are absent from the published glycosomal (58) and mitochondrial proteomes (59), but were also not identified in a *T. brucei* cell surface proteome (60). Members of the AAT7 locus were found in the membrane proteome of the trypanosome flagellum (61) and in a proteome of whole flagella (62), but the studies could not differentiate between proteins of the AAT7-A or AAT7-B subfamilies. Only one peptide unique for either 7610 or 7640 was identified (62). TrypTag showed C- and N-terminal *in situ* tagging of Tb927.8.7610 fused to mNeonGreen; however, due to the similarity between the genes it is not clear which of the AAT7-B gene copies was tagged (63). In this study tagging of the proteins at the N-terminus resulted in localization at the pellicular plasma membrane, while C-terminally tagged proteins exclusively localized at the flagellar pocket. It is possible, however, that the two localizations represent the tagging of two different proteins of the AAT7-B locus (63).

**Figure 6:**
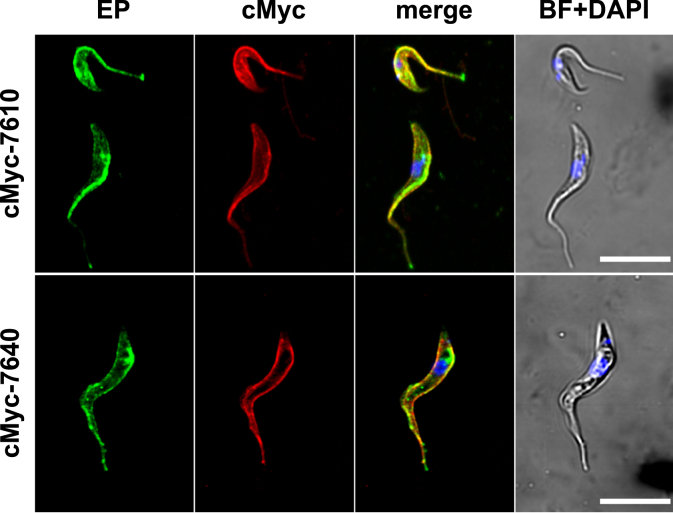
**7610 and 7640 are localized at the pellicular membrane**. PCF 29-13 cells expressing N-terminally cMyc tagged 7610 and 7640 were immunodecorated with α-cMyc antibody (*red*) and α-EP-procylin (*green*) and visualized by confocal microscopy. DAPI (*blue*), brightfield (*gray*), scale bar 10 μm.

To independently determine the subcellular localization with a smaller tag, we overexpressed 7610 and 7640 fused to an N-terminal cMyc tag in procyclic *T. brucei* cells, to allow histological staining on fixed cells for confocal microscopy. The immunofluorescence assays showed localization at the plasma membrane and partial colocalization with the plasma membrane glycoprotein marker EP procyclin (Fig. 6). We only observed a faint signal at the flagellar pocket. Localization at the plasma membrane allows the transporter to mediate uptake of proline from the environment into the cytosol of the parasite and supports cellular uptake in parasites and *S. cerevisiae*.

### Proteins encoded by the AAT7-B locus are involved in proline uptake in *T. brucei* and indispensable under conditions of proline deficiency in PCF

**Figure 7:**
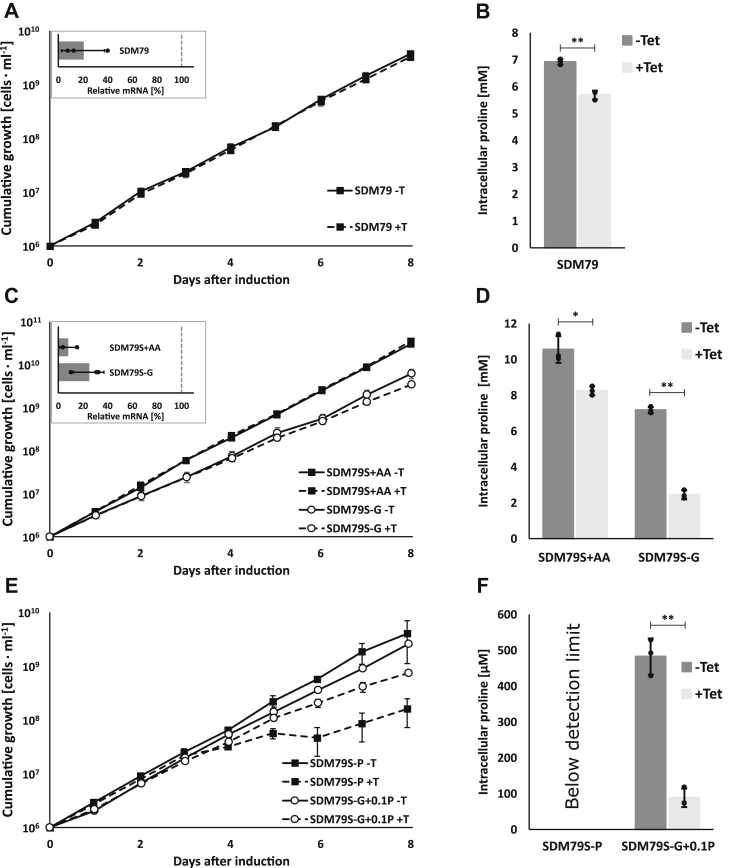
**AAT7-B RNAi in PCF *T. brucei* impacts cellular proline pools while growth is reduced only under low proline availability.***A*, growth curve in standard SDM79 ± Tet. *B*, intracellular proline concentration in cells grown for 3 days in SDM79 ± Tet as described below. *C*, growth curves of AAT7-B RNAi cells grown in starvation medium SDM79S either containing glucose (SDM79S+AA, ▪) or low glucose (SDM79S-G, ○), ± Tet. *D*, intracellular proline concentrations after 3 days of growth in SDM79S or SDM79S-G, ± Tet. *E*, growth curves of AAT7-B RNAi cells grown in starvation medium containing glucose, but with reduced proline (SDM79S-P, ▪) or in the absence of glucose and reduced proline (SDM79S-G+0.1P, ○), ± Tet. *F*, intracellular proline concentrations after 3 days of growth in SDM79S-P or SDM79S-G+0.1P, ± Tet. *A*, *C*, and *E*, growth curve (n = 3, error bars denote SD) in the presence (+T, *dashed lines*) or absence of tetracycline (−T, *solid line*). Inserts show qRT-PCR analysis of AAT7-B gene transcript levels 3 days after start of RNAi induction. Bar shows downregulation relative to noninduced cells. AN1 was used as reference gene (mean ± SD, n = 3, *dots* show individual measurements). *B*, *D*, and *F*, intracellular proline concentration in cells grown for 3 days with (+Tet) or without tetracycline (−Tet), (mean ± SD, n = 3, technical repeats, similar results were obtained in an independent biological repeat, *dots* show individual measurements). Statistical significance was determined using paired, two-tailed *t*-test (∗*p* ≤ 0.05; ∗∗*p* ≤ 0.01; n.s. not significant). Media were prepared as summarized in Table 1 and Table S1 and supplemented with 10% FBS.

To test whether the transporters are required to maintain viability of PCF *T. brucei*, we used RNA interference (RNAi) to simultaneously downregulate expression of all three AAT7-B genes. Growth in standard SDM79 medium did not result in a growth defect between induced and noninduced RNAi cells (Fig. 7*A*). The efficiency of mRNA downregulation after 3 days of RNAi induction was assessed by RT-qPCR using a common primer pair and showed 80% (±20%) reduction (Fig. 7*A*, inset). At this time point, levels of most intracellular free amino acids remained unaffected with the exception of proline, which showed ∼20% reduction in induced RNAi cells (Fig. 7*B* and Fig. S10*A*). To determine if downregulation of AAT7-B is compensated by increased expression of other amino acid transporters, we checked transcript levels of Tb927.11.15960 (AAT17.2), Tb927.8.8290 (AAT10.1), Tb927.8.8300 (AAT10.2), and Tb927.8.5450 (AAT6), but found that none of them was significantly affected (Fig. S9).

As procyclic forms have limited access to glucose *in vivo*, the performance of the RNAi cell lines was tested under low glucose conditions. For this we used the medium prepared for the starvation experiment and supplemented it with 10% (v/v) heat-inactivated FBS and glucose or GlcNAc. After adaptation of the cells to the new medium (see Materials and Methods), cells grown in SDM79S+AA (supplemented with 10% FBS, 10.8 mM glucose, and lacking GlcNAc) showed a slightly faster growth rate (doubling time ∼13 h) than cells grown in commercial SDM79 (doubling time ∼17 h) and showed no apparent growth phenotype upon RNAi induction (Fig. 7*C*). The RNAi efficiency was similar, >90% reduction of mRNA after 3 days of tetracycline addition, no change in the transcript level of other amino acid transporters (Fig. S9), and ∼20% reduction in intracellular proline levels was detected (Figs. 7*D* and S10*B*).

When cells were adapted to low glucose conditions (SDM79S-G, containing 10% (v/v) FBS and 50 mM GlcNAc), growth was slightly reduced (doubling time ∼17 h) compared with growth in SDM79S+AA with glucose, but comparable to commercial SDM79 (Fig. 7, *A* and *C*). Although cells grown in SDM79S-G medium must have an increased demand for proline uptake, a knock down did not result in a growth phenotype (Fig. 7*C*). The intracellular proline content was decreased by 66%, from ∼7.2 mM down to 2.4 mM (Fig. 7*D*), assuming a cell volume of 3.31 μl per 10^8^ cells for procyclic form cells (64). Furthermore, there was a decrease in intracellular levels of aspartate (19%) and glutamate (27%) and an increase in methionine (32%) and valine, leucine, and isoleucine (∼18%) (Fig. S10).

To further challenge the cells and test for proline auxotrophy reported by previous studies (13, 14), we tested growth under conditions of reduced proline. The main source of proline in SDM79 can easily be modified while proline added through FBS was kept constant and was estimated to account for 130 μM proline in the final medium (30, 65). Omitting proline and glucose and growing cells in SDM79S with 10% FBS and 50 mM GlcNAc (SDM79S-G-P) led to growth arrest 6 days after transfer to this medium (n = 1, Fig. S11). This confirmed proline auxotrophy in low glucose conditions but did not allow us to study the role of the proline transporters.

To enable stable growth, the addition of 530 μM proline was required, corresponding to 10% of proline present in standard SDM79 (named SDM79S-G+0.1P). This medium supported stable cell growth for at least 4 weeks; longer periods were not tested. In AAT7-B RNAi cells stably growing in SDM79S-G+0.1P, we found 500 μM proline, which is already five times lower than during downregulation of AAT7-B in cells grown in SDM79S-G (Fig. 7, *E* and *F*). Knock-down of the proline transporters under these conditions further decreased internal proline to 90 μM after 3 days of RNAi induction (Fig. 7*F*), accompanied by a growth defect starting on day 5 (Fig. 7*E*). Further changes were a decrease in the levels of Asp and Glu by 25% and 32%, respectively, increases in Val, Leu and Ile by 16–18%, and a strong increase in methionine levels by 78% (Fig. S10).

We further tested the requirements for proline under conditions where it is not needed for energy production. To date, no additional function for proline, other than protein synthesis, is known when glucose is present. It was shown previously that log-phase PCF grown in SDM80 containing both glucose and proline has a proline consumption rate of approximately 30–50 pmol min^−1^ 10^6^ cells^−1^ (14), which would correspond to 1–2% of the proline provided by SDM79. We prepared SDM79S containing 10.8 mM glucose, no proline, and supplemented it with 10% (v/v) FBS (SDM79S-P), which should result in a final proline concentration of approximately 130 μM. Noninduced RNAi cells adapted to this medium showed slightly reduced growth rates compared with cells grown with 5.47 mM proline (Fig. 7, *C* and *E*). The proline content in these cells was so low that it was below the detection limit (Fig. 7*F*). Knock-down of the transporters led to a growth defect starting after 4 days of RNAi induction (Fig. 7*E*). This again was accompanied by an increase in methionine levels by about 40% while under these conditions aspartate was increased by over 100% (Fig. S10).

**Figure 8:**
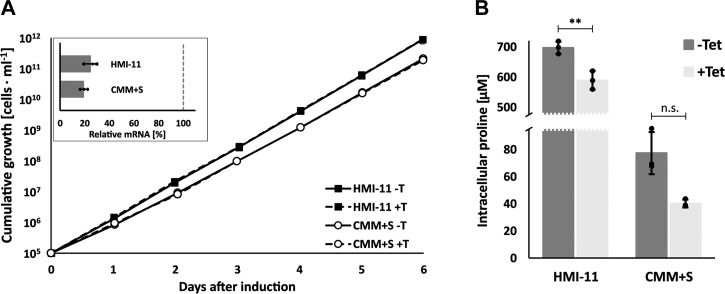
**RNA interference against AAT7-B in BSF *T. brucei*.***A*, growth curve of RNAi cells in HMI-11(▪) or CMM+S (○), with (+T, *dashed line*) or without tetracycline (−T, *solid line*) (mean ± SD, n = 3). Insert shows qRT-PCR analysis of AAT7-B RNAi in HMI-11 and CMM+S, RNA was extracted 2 days after start of RNAi induction. Bars show downregulation in cells grown in the respective medium relative to noninduced cells. TERT was used as reference gene (mean ± SD, n = 3, *dots* show individual measurements). *B*, intracellular proline concentrations after 2 days of growth in HMI-11 or CMM+S, with (*light gray column*) or without tetracycline (*dark gray column*) (mean±SD, n = 3, *dots* show individual points). Statistical significance was determined using paired, two-tailed *t*-test (∗*p* ≤ 0.05; ∗∗*p* ≤ 0.01; n.s. not significant).

To investigate the role of the transporters in the bloodstream stage, we generated an RNAi cell line in NY-SM cells. In the bloodstream stage, the parasite generates energy through glycolysis, and proline is only required for protein synthesis. The standard medium for bloodstream form culture, HMI-11, contains 347 μM proline and a further ∼130 μM proline from the serum component (50). In this medium induction of RNAi did not lead to any growth defect (Fig. 8*A*). mRNA was reduced by ∼80% after 2 days of tetracycline induction (Fig. 8*A*), but intracellular proline was only reduced by 15%, leaving a residual proline concentration of 600 μM (Figs. 8*B* and S12*A*). As already shown in the procyclic form, 500 μM intracellular proline (in SDM79S-G medium) did not lead to any growth defect, so the remaining 600 μM in the BSF cells is expected to be high enough to cover demands for protein synthesis. To reduce proline in the growth medium, we used an alternative medium, CMM, which allows amino acid concentrations to be manipulated. CMM was supplemented with 10% (v/v) heat-inactivated FBS (standard quality, Gibco) in place of the originally suggested FBS Gold. A slight growth defect for NY-SM cells could be ameliorated by addition of 100 μM each of tyrosine, phenylalanine, tryptophan, leucine, methionine, arginine, and hypoxanthine, as recommended in the original publication (65) (CMM+S). Cells adapted to this medium showed a reduction of intracellular proline levels to 80 μM compared with ∼700 μM for cells grown in HMI-11 (Fig. 8*B*). Upon knock-down of the transporters in cells grown in CMM+S, proline levels decreased to 40 μM, but the cells did not exhibit a growth defect (Fig. 8, *A* and *B* and Fig. S12*B*). Furthermore, AAT7-B downregulation had no effect on transcript levels of Tb927.8.8290 (AAT10.1), Tb927.8.8300 (AAT10.2), or Tb927.8.5450 (AAT6), in either of the two media tested, while Tb927.11.15960 (AAT17.2) showed a small decrease during knock-down in CMM+S (Fig. S13).

Unfortunately, a further reduction of proline concentration in CMM+S was not possible, as FBS is essential for prolonged growth. Therefore, the role of the transporters at very low proline concentrations, for example, 3 μM, the concentration of proline in the cerebrospinal fluid, could not be tested. Furthermore, we cannot exclude that the remaining protein left due to incomplete knock-down by RNAi is sufficient to provide for the cells' demand for proline. Therefore, we decided to generate a knockout cell line for the AAT7-B proline transporters. Because of the sequence homology and substrate similarity between the AAT7-B genes, we decided to knock out the entire locus containing the tandem repeat of the transporter genes. The first attempt to knock out the transporters by an allele replacement approach using antibiotic cassettes supported by CRISPR-Cas9-induced double-stand breaks at the beginning and end of the locus (66) did not result in any viable cells after transfection, nor in cells with incomplete knockout due to an alternative allele replacement. The second approach used an inducible expression of sgRNA, which should cut in the ORF of the transporter genes and introduce a loss-of-function mutation due to microhomology-mediated joining (67). This also failed, because all viable cell lines that we tested avoided the knockout by introducing a silent mutation at the sgRNA recognition site, thereby preventing the generation of double-strand breaks.

## Discussion

Sensing and adaptation to new environments and nutrients are driving forces for evolution and key aspects of survival for every organism including parasites. This seems particularly important for *T. brucei* considering the massive and rapid changes encountered when changing host. By using transcriptome analyses, we monitored the response of *T. brucei* to amino acid and glucose starvation and found that several amino acid transporters were highly upregulated, among them all genes of the amino acid transporter AAT7-B subgroup. Functional characterization of Tb927.8.7610 (Tb427_080081900), Tb927.8.7630 (Tb427_080082000), and Tb927.8.7640 (Tb427_080082100) showed that AAT7-B members represent plasma membrane-localized, high- and low-affinity proline/alanine transporters that are essential under low proline availability.

Previous studies showed that shifting procyclic form *T. brucei* from glucose-rich to glucose-free medium leads to a upregulation of proline catabolism and a concomitant increase in proline uptake (14). Consistent with these findings, higher expression of Tb927.8.7610, Tb927.8.7630, Tb927.8.7640 as well as of genes involved in proline catabolism was shown in PCF *T. brucei* depleted of glucose. This suggests that AAT7-B expression is connected to a switch in energy metabolism. While AAT7-B genes are expressed in all developmental stages, transcript levels of the individual genes were found to differ between BSF and PCF in several studies; these consistently showed a prevalence of Tb927.8.7610 in BSF and Tb927.8.7640 in the procyclic form (17, 39, 41, 42, 43, 44, 45, 46, 68). This is comparable to our results with approx. sixfold higher transcript levels of Tb927.8.7610 in BSF *versus* PCF and 1.5-fold more Tb927.8.7640 in BSF *versus* PCF cells under standard *in vitro* growth conditions.

It was found that short stumpy BSF preadapts to life in their future insect host (69), for example, by increasing expression of enzymes of the TCA cycle, change of their excreted end products from pyruvate toward acetate and succinate (39, 69, 70, 71), and by elevating transcripts of proline-metabolizing enzymes (17, 39, 41). Though higher AAT7-B transcript or protein levels were occasionally identified in short stumpy BSF (17, 72), in the majority of studies no major regulation was reported (41, 45, 46, 68) indicating that AAT7-B expression may be altered only once stumpy forms enter the insect host *i.e.*, during the transition to procyclic forms and induced by nutrient or energy availability.

The transition of bloodstream to procyclic forms *in vitro* can be induced by different stimuli such as a drop in temperature, addition of citrate/cis-aconitate, mild acid, or proteases (25, 73, 74, 75, 76). Depleting BSF of glucose also induced this transition as well as upregulation of enzymes involved in proline metabolism (17). Consistent with this, inhibition of the glycolytic flux by addition of 2-deoxy-glucose to BSF cells leads to the upregulation of proline dehydrogenase and also of citrate synthase (77). Moreover, addition of phloretin, an inhibitor, or glucose transporters causes at least partial differentiation of BSF to PCF (77). Glucose deprivation seems to be a rather slow adaptation and differentiation signal (76); nevertheless, it may be sufficient to induce AAT7-B expression.

A recent study investigated that the effect of suramin treatment on BSF showed that suramin decreases cytosolic ATP levels and causes a concomitant increase in proline metabolism and partial citric acid cycle activation (78). Together with enzymes of proline metabolism and oxidative phosphorylation, the AAT7-B transporters where identified among the top ten proteins upregulated after prolonged suramin treatment (78). This represents a scenario where glucose is present, which theoretically should suppress proline metabolism (14), but cytosolic ATP is decreased, arguing for energy rather than glucose-dependent regulation of AAT7-B. However, suramin has diverse effects, which make it difficult to interpret these results conclusively.

Upregulation of AAT7-B and Tb927.11.15960 mRNA was also detected under selective proline starvation. The function of Tb927.11.15960, which was among the most highly upregulated transporters, and the proline-starvation response need to be explored in future studies. At what point cells sense proline starvation is unclear, given that as little as 530 μM, which is 10% proline present in standard medium, is sufficient to sustain growth at a normal rate. Proline availability in the fly is rather controversial; reported hemolymph concentrations range from 1 to 150 mM (11, 12, 79). Moreover, during hunger cycles or flight, proline levels can be 4–5 times lower than in resting flies (11, 26, 80). It is also poorly understood to what extent proline is accessible to parasites in the various organs and compartments of the fly. A single study investigated that proline concentrations in the midgut and hemolymph of tsetse flies suggest that hemolymph reflects the amino acid concentrations available to *T. brucei* at least in the gut (12).

Increased expression of Tb927.8.7610 and Tb927.8.7640 can be observed *in vivo*. Both transcripts are higher in the proventriculus than in the fly midgut, and they are further upregulated in the salivary glands (81). Proline concentrations in saliva might be very low as first studies failed to detect proline or only found minute amounts (82, 83). This would call for the expression of a high-affinity proline transporter in *T. brucei* inhabiting the salivary gland. It has, however, also been shown that *T. brucei* can influence the composition of tsetse fly saliva and change the flies' feeding behavior (84), therefore the composition of saliva from infected flies may differ from noninfected flies. The high-affinity transporter Tb927.8.7610 was found at significantly higher levels in salivary glands than the low-affinity AAT7-B transporters (81). This might help with the uptake of trace amounts of proline and might also prepare cells for their transfer to the mammalian host. However, once salivary gland epimastigotes undergo metacyclogenesis, AAT7-B transporter expression is reduced again (85).

Proline uptake by the AAT7-B transporters measured in *S. cerevisiae* revealed that 7610 is a high-affinity proline transporter (*K*_m_ of ∼17.5 μM), while 7620, 7630, and 7640 are of low affinity (*K*_m_ 600–1000 μM). Consistent with this, Tb927.8.7610 is more highly expressed in the BSF stage, where proline levels are rather low, around 190 μM in the blood and 2 μM in the cerebrospinal fluid (30). Tb927.8.7640 is expressed ∼1.5× more in procyclic forms compared with bloodstream forms, matching increased proline levels, of 1 mM or higher in the fly (11, 12), but as mentioned before, proline levels in the fly are expected to differ in different organs.

In contrast to 7620, 7630, and 7640, which support growth on proline or cysteine, 7610 only complemented growth on cysteine. Despite these findings, subsequent short-term transport assays characterized 7610 as a high-affinity proline transporter, which recognized cysteine less efficiently. Selection during growth assays differs slightly, *i.e.*, low amounts of cysteine are required to complement the auxotrophy of *S. cerevisiae* strain Y01543, while proline was used as sole nitrogen source for *S. cerevisiae* mutant 22574d, necessitating elevated uptake to support growth. Although 7610 and 7640 differ in affinity, the apparent *V*_max_ values inferred from initial transport rates are in a similar range and thus, at first sight, cannot explain the difference in growth complementation. However, as a considerable time-dependent decline of proline uptake was observed for 7610, but not for the other transporters, (maximal) transport rates for 7610 may drop considerably in long-term experiments. Thus 7610 may only accommodate low levels of amino acid uptake in *S. cerevisiae* explaining the failure to complement growth on proline. A decline of transport rates over time has been observed for other transporters and may originate, for example, from a decrease of the inwardly directed H^+^ electrochemical gradient, or from inactivation *e.g.*, by internalization (86, 87). Whether this is also the case for 7610, when expressed in *S. cerevisiae* or in *T. brucei,* needs to be further explored.

Proline transport studies using *T. brucei* Lister 427 PCF identified a single proline transport system that shows inhibition by proline, alanine, and cysteine and an apparent affinity of 18.7 ± 1.7 μM (14, 88), but the proline concentrations used were too low to detect a possible low-affinity uptake system. The affinity is comparable with the *K*_m_ determined for 7610 expressed in *S. cerevisiae* and the main substrates were also identical. Kinetic analysis of proline uptake identified two systems in *T. cruzi* (34, 89) and three in *Leishmania* (23, 90). Only genes encoding the low-affinity components have been isolated from these parasites so far, *i.e.*, TcAAP24/TcAAAP069 and LdAAP24, respectively (23, 34). While physiological studies of the high-affinity system of *T. cruzi* showed transport of a number of amino acids, the low-affinity proline transport system was mainly inhibited by proline, cysteine, valine, and alanine (89), resembling the substrate selectivity of the TbAAT7-B transporters. TcAAP24/TcAAAP069 expressed in baker's yeast showed low affinity, but a strong preference for proline, with valine and alanine as potential substrates (34). LdAAP24 was characterized as low-affinity transport system that preferentially recognizes proline and to a lesser extent alanine (23). Additionally a shorter isoform, generated by alternative splicing, is selective for proline (91). In *T. brucei* the transporter most closely related to TcAAP24/TcAAAP069 and LdAAP24 is the proline and eflornithine transporter AAT6, which, when expressed in heterologous systems, also supports transport of many other neutral amino acids in addition to proline (32). Thus, although having only low affinity, the substrate selectivity of TcAAP24/TcAAAP069 and LdAAP24 is more similar to AAT7-B members than to their syntenic gene in *T. brucei*. As reported for LdAAP24, alanine is also a substrate for AAT7-B when expressed in yeast. Alanine makes up a big portion of the free amino acid pool in trypanosomes (21, 92, 93, 94) and was shown to be involved in osmoregulation in *L. donovani* and *T. cruzi* (23, 24), though this has not been reported yet in *T. brucei*. Nevertheless, alanine is present in large quantities in the cytosol of *T. brucei* (21, 92, 94) and represents an end product of proline metabolism in PCF (16, 95). Alanine cannot be further metabolized to CO_2_ as in *T. cruzi* (96), and therefore needs to be exported in PCF *T. brucei*. While in *T. brucei* BSF the major end product of glycolysis is pyruvate (65, 94, 95), alanine is also excreted in moderate amounts (65, 94), and elevated alanine levels have been found in the serum of infected rats (97) or voles (98), which suggests that alanine is being exported from the parasite. Therefore, under isotonic conditions, alanine export rather than import is expected. However, as intracellular alanine showed only minor changes in the AAT7-B knock-down lines, it seems rather unlikely that AAT7-B members mediate alanine export.

Multiple transporters can help to fine-tune proline uptake according to the current requirements, and transporters may differ in activity depending on the environment of the parasite. It was therefore not surprising to find more than one proline transport system in *T. brucei.* Because trypanosomatids rely on proline catabolism in their insect hosts, it seemed likely that not only proline catabolism genes, but also proline transporters would be conserved/syntenic over species (8). It was therefore interesting to find transporters belonging to a very different gene locus in *T. brucei*, compared with *Leishmania* or *T. cruzi*, to be involved in the uptake of proline. Multiple uptake systems can also form redundant backup systems, but at least in our RNAi experiment under low proline conditions, AAT6 could not sustain cell growth. This might be due to the low affinity of AAT6 for proline and/or its broad substrate specificity (32). RNAi knock-down in PCF also did not lead to a growth defect under low glucose conditions. Only a combination of AAT7-B knock-down together with low proline concentration resulted in a growth defect, so AAT6 transport activity might compensate at high proline concentrations, though AAT6 expression was not elevated in the RNAi lines. We also checked for transcript levels of transporters that were regulated during short-term glucose or amino acid starvation, but found none of them affected by knock-down of the AAT7-B transporters. This also fits the observation that none of these transporters, except Tb927.11.15960, showed any response to selective proline starvation, which we expect to be the main effect of the knock-down of AAT7-B transporters in *T. brucei.* Tb927.11.15960 transcript levels were increased during proline starvation, but under the conditions tested, the reduction of intracellular proline in the AAT7-B RNAi lines was probably too low to increase Tb927.11.15960 transcript levels. Based on these data, compensation of proline uptake by these transporters during AAT7-B-RNAi is not supported, but of course we cannot exclude upregulation of other transporters that did not show up in our initial RNAseq experiment or regulation at the translational or posttranslational level. Although we cannot rule out the possibility that there are additional proline transporters besides AAT7-B and AAT6, the response of AAT7-B members during starvation, in parallel with genes encoding enzymes of proline catabolism, strongly indicates that the transporters of the AAT7-B family are indeed the main proline transporters in PCF.

## Experimental procedures

### Plasmid constructs

For heterologous expression in *S. cerevisiae*, open reading frames (ORFs) of the genes Tb927.8.7610 (Tb427_080081900), Tb927.8.7630 (Tb427_080082000), and Tb927.8.7640 (Tb427_080082100) were PCR amplified from genomic DNA of *T. b. brucei* Lister 427 using PfuUltra DNA polymerase AD (Stratagene) and primers 5′-CGGAATTCATGACCAGCATCAATGCCCAACC-3′ and 5′-CGCGGATCCTCATCCCACAGTAACTGCCCAAATGG-3′, and cloned into the yeast expression vector pDR197 (99) using the *Eco*RI and *Bam*HI restriction sites, resulting in construct 7610 (for Tb927.8.7610), 7630 (for Tb927.8.7630), 7640 and 7640′ (for Tb927.8.7640). The construct 7630 was mutated by two rounds of site-directed mutagenesis PCR using first primers 5′-CCTATCTTTTTCAGATTACCGCGTATG-3′ and 5′-CATACGCGGTAATCTGAAAAAGATAGG-3′, to give construct 7630′, and in a second step primers 5′-CGTTCTGGTTGTCACCATTGCGATGGG-3′ and 5′-CCCATCGCAATGGTGACAACCAGAACG-3′ to obtain construct 7620, coding for an identical amino acid sequence as for Tb427.08.7620 described in TriTrypDB.

The construct required for the simultaneous knock-down of all genes of the AAT7-B locus was generated by amplifying a 390 bp DNA fragment spanning the 3′end of the ORF and the beginning of the 3′UTR using genomic DNA of *T. b. brucei* Lister 427 and primers 5′-CCGGAAGCTTGGATCCAAAGGTTGGTCCCTTTTATTACATTTCC-3′ and 5′-TGGCTCTAGACTCGAGCGACACGAACAAAGGAAATAATTGAC-3′. The fragment was cloned into the stem-loop vector pALC14 (a derivative from pLew100 (20, 48, 100), containing a puromycin resistance), in two steps, inserting the first fragment using the *Xho*I and *Bam*HI and the second into *Hind*III and *Xba*I.

For inducible overexpression of tagged versions of Tb927.8.7610 and Tb927.8.7640, the ORFs of construct 7610 and 7640 were cloned in the plasmid pJM-2 (based on pLew100 (48) containing a puromycin resistance and a 3x cMyc cassette for N-terminal tagging (101)), creating the plasmids cMyc-7610 and cMyc-7640, respectively.

### *S. cerevisiae* strains, growth conditions, and transport assays

*S. cerevisiae* was transformed according to Dohmen *et al.* (102). To test growth on different amino acids, ORFs were expressed in the following *S. cerevisiae* mutants: 21.983c (Matα, gap1-1, can1-1, ura3) (103) for arginine, 22574days (Matα, ura3-1, gap1-1, put4-1, uga4-1) (104) for citrulline, γ-aminobutyric acid and proline, 22Δ6AAL (Matα, ura 3–1, gap1-1, put4-1, uga4-1, lyp1/alp1::hisG, can1::hisG, hip1:hisG) (105) to test for lysine, 30.537a (Matα, gap1-1, dip5::kanMX2, ura3; kind gift of Professor Bruno André, Université Libre de Bruxelles) for glutamate and aspartate, JT16 (Matα, hip1-614, his4-401, can1, ino1, ura3-52) (106) for histidine, Y01543 (Matα, his3Δ1, leu2Δ0, met15Δ0, ura3Δ0, YLL055w::kanMX4; Euroscarf) for cysteine, and YDR544 (Matα, ura3-1, gap1-1, put4-1, uga4-1, ssy1::kanMX) (107) for the amino acids methionine, valine, isoleucine, leucine, threonine, tryptophan, and tyrosine. Nonselective and selective conditions were: for mutant 21.983c, synthetic minimal glucose medium (MM, 1.7 g L^−1^ yeast nitrogen base without amino acids and without ammonium sulfate (Difco), and 20 g L^−1^ glucose) containing 5 g L^−1^ (NH_4_)_2_SO_4_, or in minimum buffered (pH 6.1) medium (M.am, without ammonium sulfate, (108)) containing 1 g L^−1^ arginine instead of (NH_4_)_2_SO_4_; for mutant 22574d, MM containing 5 g L^−1^ (NH_4_)_2_SO_4_, or M.am medium containing either 1 g L^−1^ proline, 1 g L^−1^ γ-amino butyric acid, or 1 g L^−1^ citrulline; for mutant 22Δ6AAL, MM containing 1 g L^−1^ urea and 1 g L^−1^ lysine or 100 μM lysine; for mutant 30.537a, MM containing 5 g L^−1^ (NH_4_)_2_SO_4_, or M.am medium containing either 1 g L^−1^ glutamate, or 0.5 g L^−1^ aspartate; for mutant JT16, synthetic complete (SC, without histidine (109)) medium containing either 20 mM or 5 or 6 mM histidine; for mutant Y01543, MM containing 50 mg L^−1^ leucine, 50 mg L^−1^ histidine and 50 mg L^−1^ methionine, or M.am containing 10 mM (NH_4_)_2_SO_4_, 50 mg L^−1^ leucine, 50 mg L^−1^ histidine and 100 μM cysteine; and for mutant YDR544, MM containing 5 g L^−1^ (NH_4_)_2_SO_4_, or MM containing either 1 mM isoleucine, 5 mM leucine, 1 mM methionine, 5 mM threonine, 1 mM tryptophan, 1 mM tyrosine, or 1 mM valine. As positive controls for *S. cerevisiae* growth on aspartate, glutamate, and lysine, we used a construct for the expression of the amino acid transporter AAP6, of *Arabidopsis thaliana* (AtAAP6, (105)), for arginine the arginine transporter, AAP3, of *L. donovani* (LdAAP3, (107)), for γ-amino butyric acid the high-affinity GABA transporter, GAT1, of *A. thaliana* (AtGAT1, (110)), for cysteine the high-affinity cysteine transporter from *S. cerevisiae* (ScYct1, (111)), and for all other amino acids the general amino acid permease AAP2 of *A. thaliana* (AtAAP2, (112)).

Transport assays were performed as described before (113) with slight modifications. Transformed *S. cerevisiae* mutant 22574d was grown in MM containing 5 g L^−1^ ammonium sulfate to an OD_578_ of 0.6, Y01543 was grown in MM containing 5 g L^−1^ ammonium sulfate, 50 mg L^−1^ leucine, 50 mg L^−1^ histidine, and 50 mg L^−1^ methionine. To reduce transport by endogenous transport systems, cells were cultivated for 1 h in yeast extract peptone dextrose (YPD) medium, before cells were harvested by centrifugation. Cells were washed once with water, diluted to an OD_578_ of 0.6, pelleted and resuspended in 1/10 volume of buffer A (0.6 M sorbitol, 50 mM potassium phosphate, pH 7.0), and placed on ice. For determination of pH dependence of proline uptake, buffer A was adjusted to pH 4.5, 5.0, 5.5, 6.0, 6.5, 7.0, or 7.5. Prior to starting the transport assay, cells were incubated for 5 min at 30 °C in the presence of 100 mM glucose. To start the assay an equal volume of cell suspension and substrate were mixed and incubated at 30 °C. Substrate mixtures contained varying concentrations of nonlabeled proline spiked with L-[2,3,4,5-^3^H]-proline (MT-522, Hartmann Analytics, specific activity 2.1 TBq mmol^−1^) to a final activity of 55.5 kBq ml^−1^ and competitor compounds as specified. For measurement of alanine uptake, nonlabeled alanine was spiked with L-[2,3-^3^H]-alanine (MT-866, Hartmann Analytics, specific activity 1.41 TBq mmol^−1^) to a final activity of 55.5 kBq ml^−1^. For cysteine transport, nonlabeled cysteine was spiked with L-[^35^S]-cysteine (ARS0101, Hartmann Analytics, specific activity 39.8 TBq mmol^−1^) to a final activity of 107 kBq ml^−1^ and 250 μM DTT was added. After 15, 30, 45, 60, and 75 s (for 7610, unless stated otherwise) or 30, 60, 120, 180, and 300 s (for 7620, 7630, and 7640), aliquots were transferred to 4 ml ice-cold buffer A, filtered on glass microfiber filter (Whatman grade GF/C), and washed twice with 4 ml chilled buffer A. The filters were immersed in 4 ml Ultima Gold XR (PerkinElmer) scintillation cocktail and samples were analyzed by liquid scintillation counting on a Tri-Carb 2910 TR (PerkinElmer). Results are displayed as uptake rates calculated from the time series performed in each transport assay. As background, transport rates measured in cells transformed with the yeast expression vector were subtracted from the sample values. Calculation of kinetic parameter of the transporters was performed using nonlinear least squares regression using the Michaelis–Menten kinetic model.

### *T. b. brucei* cell culture

Procyclic *T. brucei* 29-13 cells (48), SmOxP9-Cas9 cells (66), and thereof derived cells were routinely cultured at 27 °C in standard medium SDM79 (49) (purchased from BioConcept, Switzerland) or in a home-made medium SDM79S (containing low glucose, low proline, and/or low amino acid concentrations, see Table 1, for detailed composition Table S1) *i.e.*, for starvation experiments.

All media were supplemented with 7.5 mg L^−1^ hemin and 15 mg L^−1^ folic acid (as hemin-folate solution). For continuous cultivation and growth curves, media were further supplemented with 10% (v/v) heat-inactivated fetal bovine serum (FBS) (Gibco). Depending on cell line, PCF cells were grown with 50 μg ml^−1^ hygromycin, 15 μg ml^−1^ neomycin, 2.5 μg ml^−1^ phleomycin, 10 μg ml^−1^ blasticidin, and/or 1 μg ml^−1^ puromycin, as required.

Bloodstream form *T. brucei* NY-SM (48), SmOxB4-Cas9 cells (66), and derived cell lines were cultured at 37 °C under 5% CO_2_ in medium HMI-11 (50), in home-made medium HMIS (containing low amino acids or low glucose, see Table 1, for detailed composition Table S1), or in Creek's minimum medium (CMM), supplemented with tyrosine, phenylalanine, tryptophan, leucine, methionine, arginine, and hypoxanthine, each at 100 μM (CMM+S) (65). For continuous growth HMI-11 and CMM were prepared with 10% (v/v) heat-inactivated FBS (Gibco) as serum component. BSF cells were grown in the presence of 5 μg ml^−1^ hygromycin, 1.25 μg ml^−1^ phleomycin, 5 μg ml^−1^ blasticidin, and/or 0.1 μg ml^−1^ puromycin, as required.

Cells were subcultured every 24 h to keep them at cell densities of 0.1–1.5 × 10^7^ cell ml^−1^ for procyclic *T. brucei* cultures and 0.1–2 × 10^6^ cells ml^−1^ for BSF trypanosomes. For growth curves cells were grown in a volume of 1 ml in 24-well plates, diluted every day to the same starting concentration, and for induction of RNAi tetracycline was added at a concentration of 1 μg ml^−1^.

For adaption of cells to new media (*e.g.*, low glucose, or low proline medium for PCF or CMM for BSF), cells were diluted from a standard log-phase culture in standard medium (commercial SDM79 or standard HMI-11) with new medium and grown for 3–4 days with daily dilutions with fresh media conditions, so cells could multiply at least 100-fold, allowing a 1:100 dilution of the old media and adaption of a stable growth, before start of any experiment. For adaption of PCF to low glucose–low proline medium (SDM79S-G+0.1P), cells were first adapted to low glucose medium (SDM79S-G) and then adapted to SDM79S-G+0.1P.

### Transfection of trypanosomes

Plasmid DNA for generation of RNAi cell lines or for expression of tagged proteins was linearized by *Not*I and purified using the NucleoSpin Gel and PCR Clean-up kit (Macherey-Nagel). For transfection 1 × 10^7^ cells of a BSF NY-SM or PCF 29-13 mid-log culture were harvested by centrifugation at 800*g*. After complete removal of the supernatant, the cell pellet was resuspended in 100 μl Tb-BSF buffer (90 mM Na_2_HPO_4_, 5 mM KCl, 0.15 mM CaCl_2_, 50 mM HEPES, pH 7.3, (36)) containing 10 μg of linearized plasmid DNA, and electroporated using a Lonza 4D-Necleofector System (Primary Cell P3, pulse code FI-115). Pulsed cells were transferred to 10 ml fresh medium for 12–16 h. After recovery cells were further diluted 1:5 and 1:25 and PCF cells were spread onto a 48 well plate (0.4-2 × 10^5^ cells well^−1^), while BSF cells were spread on 96 well plates (0.4–2 × 10^4^ cells well^−1^). For selection and generation of clonal populations, standard medium was supplemented with 20% FBS and selective antibiotics were added at the following concentrations, for PCF, 50 μg ml^−1^ hygromycin, 30 μg ml^−1^ neomycin, 5 μg ml^−1^ phleomycin, and/or 2 μg ml^−1^ puromycin; for BSF, 5 μg ml^−1^ hygromycin, 5 μg ml^−1^ neomycin, 2.5 μg ml^−1^ phleomycin, and/or 0.2 μg ml^−1^ puromycin, as required.

### CRISPR-Cas9 knockout generation in *T. brucei*

For generation of AAT7-B knockout cell lines by allelic replacement with the help of CRISPR-Cas9, sgRNA flanking the AAT7-B locus was designed with the help of EuPaGDT web tool (114), and PCR and *T. brucei* transfection were performed following the published protocol (66). The guide RNA template containing a T7 promoter for *in vivo* transcription was generated by PCR using for the 5′-sgRNA the primer 5′-GAAATTAATACGACTCACTATAGGAGCTGGGCAAGCGAATGCTG-3′ 5′-GTTTTAGAGCTAGAAATAGC-3′ and for the 3′-sgRNA the primer 5′-GAAATTAATACGACTCACTATAGGTGGAGCGAACGAATGAGAGAGTTTTAGAGCTAGAAATAGC-3′, together with the guide RNA scaffold primer 5′-AAAAGCACCGACTCGGTGCCACTTTTTCAAGTTGATAACGGACTAGCCTTATTTTAACTTGCTATTTCTAGCTCTAAAAC-3′. Resistance markers for replacement of both alleles were amplified for hygromycin resistance from the plasmid pPOTv7-HYG-mNG and for neomycin resistance from pPOTv7-G418-mNG (115), using the primers 5′-CAACATTCTTACTTTCCCCTTACCTCATATTTTCTATATCCATCAAGTGTGTATAATGCAGACCTGCTGC-3′ and 5′-ACAAAATAAAATAACGTTTTGGAGAGGGGAACCGCGCTTGAAATACGTGTCCGGAACCACTACCAGAACC-3′. All PCR reactions were pooled and cleaned using the NucleoSpin Gel and PCR Clean-up kit (Macherey-Nagel), and cleaned DNA was used for the transfection of PCF SmOxP9-Cas9 or BSF SmOxB4-Cas9 cells. Transfection and selection of cells were performed as described above. Viable clones were tested by PCR for integration of both resistance markers and for the loss of all AAT7-B genes.

For generation of inducible CRISPR-Cas9 knockout cell lines (67), a guide RNA was designed, which induced a double-strand break the ORF of all genes of the AAT7-B locus. For this the oligos 5′-AGGGACCACTGCTGTTGGCGGCTGC-3′ and 5′-AAACGCAGCCGCCAACAGCAGTGGT-3′ were annealed to each other and cloned into the plasmid pT7^sgRNA^ (67). The plasmid was linearized with *Not*I and used for transfection of PCF SmOxP9-Cas9 and BSF SmOxB4-Cas9 cells, with the transfection protocol as described above. Following selection, sgRNA expression was induced by addition of 1 μg ml^−1^tetracycline to the media for 7 days. After the induction period, the ORFs of the AAT7 genes were amplified by PCR and sequenced to confirm microhomology repair events of the CRISPR-Cas9-induced double-strand breaks.

### Starvation experiments and RNA extraction

For starvation experiments, mid-log cultures of PCF 29-13 cells were grown in SDM79 with 10% FBS. 5 × 10^7^ cells were harvested by centrifugation at 800*g* for 10 min at room temperature, followed by one wash with an SDM79S base (SDM79S without amino acids or glucose), containing no amino acids, no glucose (detailed composition in Table S1). Cell pellets were resuspended in 10 ml fresh starvation medium prewarmed to 27 °C. Media used were either commercial SDM79 containing 10% (v/v) FBS (named SDM79+FBS, or FBS for the starvation experiment) or the starvation medium SDM79S containing glucose and all standard amino acids (Ala, Arg, Asn, Asp, Cystine, Glu, Gln, Gly, His, Ile, Leu, Lys, Met, Orn, Phe, Pro, Ser, Taurine, Thr, Trp, Tyr, Val) (SDM79S+AA, or +AA), SDM79S containing glucose but no amino acids (SDM79S-AA, or -AA), SDM79S containing no glucose, but all amino acids and 50 mM N-acetyl glucosamine (SDM79S-G, or -G). For investigation of the selective effect of proline during starvation, further media were prepared, SDM79S containing glucose and all amino acids with the exception of proline (SDM79S-P, or -P) and SDM79S containing glucose and 5.3 mM proline, but no other amino acids (SDM79S-AA+P, or -AA+P). None of the media used for the starvation experiments, except SDM79+FBS, was prepared with FBS. For a summary of media compositions, see Table 1, and for detailed composition, see Table S1.

For starvation experiments using BSF NY-SM cells, cells were grown to mid-log phase in HMI-11, and 5 × 10^7^ cells were harvested by centrifugation at 800*g* for 10 min at room temperature, followed by one wash with HMIS salt base, containing glucose, but no amino acids, and no vitamins (detailed composition in Table S1). Cell pellets were resuspended in 50 ml fresh, prewarmed starvation medium and incubated at 37 °C with 5% CO_2_. Media for BSF starvation were HMI-11 containing 10% (v/v) FBS (HMI-11, or FBS), the starvation medium HMIS containing glucose and amino acids (HMIS+AA, or +AA), HMIS containing glucose, but no amino acids (HMIS-AA, or -AA), and HMIS containing amino acids but no glucose (HMIS-G, or -G). None of the starvation media, except HMI-11, was prepared containing FBS. The overview of all media used is summarized in Table 1 and detailed media compositions are summarized in Table S1.

For the starvation period cells were kept in 15 ml (PCF) or 50 ml (BSF) Falcon tubes with open lids, and cells were mixed by inversion every 30 min. Cells were starved for 2 h, 4 h, or 6 h, followed by RNA extraction using guanidium thiocyanate (116). In short, cells were harvested by centrifugation and washed once with phosphate buffered saline (PBS) followed by lysis of the cells with solution D (4 M guanidine thiocyanate, 25 mM sodium citrate pH 7.0, 0.5% (v/v) sodium lauroyl sarcosinate, 0.1 M 2-mercaptoethanol). The cell lysate was extracted with acidic phenol-chloroform-isoamyl alcohol, 150:49:1, (v/v/v) (using ROTIAqua-Phenol for RNA extraction, Carl Roth, CH) and RNA from the aqueous phase was precipitated with ethanol. The RNA was treated with DNase I followed by an additional acidic phenol extraction and ethanol precipitation.

For assessment of RNAi efficiency of RNAi cell lines, cells were grown in medium with or without 1 μg ml^−1^ tetracycline for 2 days (BSF), or 3 days (PCF), and RNA was extracted with the SV Total RNA Isolation System (Promega).

### RNAseq and data analysis

RNA extracted from the starvation experiment using guanidine thiocyanate (116) was sent to Fasteris SA (Geneva, Switzerland) for RNAseq analysis. Raw RNA was poly-A purified and cDNA libraries were prepared using the Ilumina TruSeq stranded mRNA kit. Sequencing was performed using Ilumina NextSeq with single-reads at 150 bp size, or Illumina HighSeq with paired reads at 150 bp size. Sequencing depth averaged >20 million reads per sample.

Reads were mapped to all transcripts of *T. b. brucei* Lister 427 (51) (TriTrypDB release 46; referred to as Lister 427_2018), using the integrated bowtie tool (117) on a galaxy web server (usegalaxy.org, (118)) with default settings, allowing two mismatches within 28 bp of the seed. Read counts mapped per ORF were divided by the total number of mapped reads per sample to obtain the RPM values (reads per million mapped reads) for all identified genes. For each cell stage individually, genes were removed from the dataset, which had fewer than 100 mapped reads within the coding region of the gene, over all samples. For further refinement, all pseudogenes were removed. A separate table was generated containing all amino acid transporters including pseudogenic transcripts (included in Table S2). For all remaining identified *T. b. brucei* Lister 427_2018 genes, orthologous genes within the *T. b. brucei* TREU 927 genome were matched over identical OrthoMCL groups (119), which were extracted from TriTrypDB. Orthologues groups with multiple members that could not be uniquely matched were either noted as such and all matching 927 Gene IDs listed, or they were manually matched according to their genomic location. For assessment of gene regulation, for each gene RPM values of cells starved of amino acids or glucose were compared with RPM values of cells grown with glucose and with amino acids.

### Quantitative RT-PCR

RNA extracted from the starvation experiments or from RNAi cell lines was transcribed to cDNA using PrimeScript RT reagent kit (Takara Bio) using both oligo dT and random six mer primers. Quantitative PCR was performed using SYBR Premix Ex Taq II (Tli RNase H Plus) (Takara Bio) on a LightCycler 96 Real-Time PCR System (Roche). Primers for the simultaneous detection of the genes Tb927.8.7610 (Tb427_080081900), Tb927.8.7630 (Tb427_080082000), and Tb927.8.7640 (Tb427_080082100) were 7610F 5′-TGGACATGACAAACCGTTCG-3′ and 7610R 5′-TGGGAAGACACATACCCATCG-3′, primers for Tb927.11.15960 (Tb427_110178500) were 15960F 5′-GGCACTTTTTACGGCTTTGTTGC-3′ and 15960R 5′-CACACCTATAGAGCCTATCAGC-3′ and for Tb927.8.8300 (Tb427_080088500) were 8300F 5′-TGATGGTTGCAATGGTTGGC-3′ and 8300R 5′-TGAATTGCGAAGTGCCATGG-3′. Further genes analyzed were Tb927.8.8290 (TbAAT10.1, Tb427_080088400) (33), and Tb927.8.4710, Tb927.8.4720, Tb927.8.4730, Tb927.8.4740, Tb927.8.4750 (TbAAT5-2/3/4/5/6, Tb427_080052200, Tb427_080052300, Tb427_080052400, Tb427_080052500, Tb427_080052600) using primers published previously (31). Further primers were designed to detect the amino acid transporter Tb927.8.5450 (AAT6, Tb427_080059900), 5450F 5′-TGCGGTGTTTTTGCTTCCAG-3′ and 5450R 5′-TTCGTTCCAAGCAGCAACTG-3′. For normalization published primers for the reference genes telomerase reverse transcriptase (TERT, Tb927.11.10190, Tb427_110110500) (120) or the putative AN1-like zinc finger protein (AN1, Tb927.10.12970, Tb427_100136900) (121) were used. qRT-PCR reactions were conducted on three RNA samples from independent experiments per condition with at least two technical replicas. Statistical significance was determined using paired, two-tailed *t*-tests.

### Amino acid extraction and analysis

For analyses of intracellular metabolites from RNAi cell lines, cells were cultivated in the presence or absence of tetracycline for 2 days (BSF) or 3 days (PCF), and metabolites were extracted by organic solvent extraction (122). 5 × 10^7^ cells of a mid-log cell cultures were rapidly cooled to 0 °C in an ethanol/dry-ice bath. Cells were washed three times with ice-cold PBS (137 mM NaCl, 2.7 mM NaCl, 10 mM Na_2_HPO_4_, 1.76 mM KH_2_PO_4_, pH 7.2). The cell pellet was extracted with a mixture of chloroform, methanol, and water at a ratio of 1:3:1, by vortex shaking for 1 h at 4 °C. Subsequently the suspension was centrifuged to remove cell debris and the clear supernatant was collected and stored until analysis at –80 °C. Free amino acids were determined by ARC (Analytical Research and Services, University of Bern) based on the method of Bidlingmeyer and coworkers (123); therefore, extracts were derivatized with phenyl isothiocyanate and analysis was performed by RP-HPLC coupled to UV detection (33).

To compensate for variations in cell number represented in the final extract due to loss in the washing steps, the amount of each amino acid was normalized to the sum of all amino acids in the sample sufficiently separated during the analysis (Asp, Glu, Pro-OH, Ser, Gly, Arg, Val, Met, Ile, Leu, Lys for PCF; and Glu, Pro-OH, Asn, Ser, Gln, Gly, Arg, Tyr, Ile, Leu, Phe, Lys for BSF) excluding alanine and proline, which are the main substrates of the studied transporters. Extraction and analysis were performed as technical triplicates, and the normalized amino acid amounts were averaged between treatments (grown with or without tetracycline) followed by multiplication with the average amino acid sum over all samples (with and without tetracycline) to again obtain amino acid amounts. Assuming no loss of amino acids during the extraction and analysis, the obtained amino acid amounts were converted to intracellular concentrations assuming for PCF a cell volume of 3.31 μl 10^8^ cells^−1^ (64), and for BSF a cell volume of 5.89 μl 10^8^ cells^−1^ (64). For calculation of relative amino acid contents, following the previous normalization steps, the average amino acid amounts in cells grown without tetracycline were set to 100%. As the amount of amino acids was dependent on the growth conditions (media), some amino acids could not be sufficiently separated by HPLC to allow reliable quantification (*i.e.*, Asn, Gln, Thr), these amino acids as well as amino acids that were present at concentrations close to the detection limit (Tyr, Phe, Trp) were omitted from some of the analysis. For statistical analysis data were treated as paired samples and tested using a two-tailed *t*-test.

### Immunolocalization

For immunolocalization of N-terminal cMyc tagged ORFs of Tb927.8.7610 and Tb927.8.7640, PCF cells transfected with the tagging construct cMyc-7610 and cMyc-7640 were grown with 1 μg ml^−1^ tetracycline for 3 days to induce overexpression of the tagged proteins. Cells were harvested by centrifugation at 800*g* and washed once with PBS-G (PBS containing 10 mM glucose). Cells were fixed in solution with fresh 4% (w/v) paraformaldehyde in PBS, settled on Superfrost plus slides (Thermo Scientific), and permeabilized with 0.2% (v/v) Triton X-100 in PBS. Cells were probed with antibodies against cMyc (polyclonal, rabbit, Bethyl Laboratories) and against EP procyclin (monoclonal, mouse, CLP001AP, Cedarlane) at a dilution of 1:1000. Following the application of the secondary anti-mouse antibody conjugated with Alexa Fluor 488 (polyclonal, goat, Life Technologies) and anti-rabbit antibody conjugated with Alexa Fluor 594 (polyclonal, goat, Life Technologies), slides were mounted to coverslips with Vectashield containing 1.5 μM DAPI (Vector Laboratories). Images were recorded on a Leica DM RXE confocal microscope equipped with a Leica TCS SP2 confocal scanner. Final images were created and analyzed using Fiji (124).

## Data availability

Raw RNA-seq rea data have been deposited at the European Nucleotide Archives (ENA, http://www.ebi.ac.uk/ena) under the study accession number PRJEB40295. Individual data files are available under the accession numbers ERS5057102–ERS5057121.

## Supporting information

This article contains supporting information (31, 32, 33, 103, 105, 108, 109, 110).

## Conflict of interest

The authors declare that they have no conflicts of interest with the contents of this article.
