# Supplementary material for: Nutrient availability regulates proline/alanine transporters in Trypanosoma brucei
Source: J Biol Chem. 2021 Mar 18;296:100566. doi: 10.1016/j.jbc.2021.100566 (PMC8094907; doi:10.1016/j.jbc.2021.100566)
Supplement: Supplementary file 3 — Table S2 [file mmc3.docx]

**Table S 3**: Nucleotide changes and resulting amino acid changes of AAT7-B sequences found in sequences amplified from Lister 427 cultured in our laboratory, compared to sequences in TriTrypDB for strains TREU 927, Lister 427_2010 and Lister 427_2018. The names below the table correspond to the names used in the publication text.

**Table S 3 A** – Single nucleotide changes in genes amplified for Tb927.8.7610 from our lab strain of *T. b.*Lister 427 compared to database sequences from TriTrypDB (release 46)

|  | **Database sequences** | | | | | **Our sequences** | | | |
| --- | --- | --- | --- | --- | --- | --- | --- | --- | --- |
| **Nucleotide position in Tb927.8.7610** | **Tb927.8.7610** | **Tb427.08.7610** | **AA change** | **Tb427_080081900** | **AA change** | **7610_1** | **AA change** | **7610_2** | **AA change** |
| 5 | T | C | I to T | C | I to T | C | I to T | C | I to T |
| 47 | G | G |  | A | R to Q | G |  | A | R to Q |
| 52 | G | G |  | A | D to N | G |  | A | D to N |
| 1122 | T | C |  | C |  | C |  | C |  |
|  |  |  |  |  |  |  |  |  |  |
|  |  |  | **PCR amplicons from genomic DNA** | | | **3** |  | **1** |  |
|  |  |  | **Construct in this study** | | | **7610** |  | **-** |  |
|  |  |  |  | | |  |  |  |  |

For Tb927. 8.7610 (7610) we sequenced four independent PCR fragments, three of which corresponded to the Lister 427_2010 sequence and one corresponding to the Lister 427_2018 sequence. When compared to TREU 927 the Lister 427_2010 gene contains two point mutations, one changing isoleucine in position two to a threonine, while the sequence identical to the 427_2018 sequence contains 2 further mutation causing 2 additional amino acid substitutions. For our experiments in *Saccharomyces cerevisiae* and for overexpression in *T. brucei* we used the sequence identical to the Lister 427_2010 genome sequence.

**Table S 3 B** - Single nucleotide changes in genes amplified for Tb927.8.7620/30 from our lab strain of *T. b.*Lister 427 compared to database sequences from TriTrypDB (release 46)

|  | **Database sequences** | | | | | | | | | **Our sequences** | | | | | | | | | | | |
| --- | --- | --- | --- | --- | --- | --- | --- | --- | --- | --- | --- | --- | --- | --- | --- | --- | --- | --- | --- | --- | --- |
| **Nucleotide position in Tb927.8.7620** | **Tb927.8.7620** | **Tb927.8.7630** | **AA change** | **Tb427.08.7620** | **AA change** | **Tb427.08.7630** | **AA change** | **Tb427_080082000** | **AA change** | **7620 ^1)^** | | **AA change** | | **7630_1** | | **AA change** | | **7630_2 ^2)^** | | **AA change** | |
| 105 | G | G |  | G |  | A |  | G |  | G | |  | | G | |  | | G | |  | |
| 151 | C | T |  | C |  | T |  | C |  | C | |  | | C | |  | | C | |  | |
| 220 | G | G |  | A | V to I | G |  | G |  | A | | V to I | | G | |  | | G | |  | |
| 513 | C | C |  | C |  | C |  | T |  | T | |  | | T | |  | | T | |  | |
| 516 | T | T |  | T |  | T |  | T |  | G | |  | | G | |  | | G | |  | |
| 751 | C | T |  | C |  | T |  | T |  | T | |  | | T | |  | | T | |  | |
| 789 | A | G |  | A |  | G |  | G |  | G | |  | | G | |  | | G | |  | |
| 816 | G | G |  | G |  | G |  | T | Q to H | G | |  | | T | | Q to H | | G | |  | |
| 884 | C | C |  | C |  | C |  | T | T to I | C | |  | | T | | T to I | | C | |  | |
| 1083 | G | G |  | G |  | G |  | A |  | G | |  | | G | |  | | G | |  | |
| 1122 | T | T |  | C |  | C |  | T |  | T | |  | | T | |  | | T | |  | |
| 1159 | A | G | I to V | A |  | G | I to V | G | I to V | A | |  | | G | | I to V | | G | | I to V | |
|  |  |  |  |  |  |  |  |  |  |  | |  | |  | |  | |  | |  | |
|  |  |  |  |  |  | **PCR amplicons from genomic DNA** | | | | **-** | |  | | **2** | |  | | **-** | |  | |
|  |  |  |  |  |  | **Construct in this study** | | | | **7620** | |  | | **7630** | |  | | **7630’** | |  | |
|  |  |  |  |  |  | ^1)^ 7620 was generated by mutation of 7630_2 ^2)^ 7630_2 was generated by mutation of 7630_1 | | | | |  | |  | |  | |  | |  | |  |

During the amplification of Tb927.8.7630 (7630) two independent PCR products coding for an identical amino acid sequence as the Lister 427_2018 gene (Tb427_080082000) were cloned. The sequence contains three amino acid changes to the database sequences of Tb927.8.7630. We did not amplify any ORF which would correspond to the Lister 427_2010 sequence of Tb927.8.7620 (7620), which is also absent in the Lister 427_2018 genome assembly. Therefore the Tb427.08.7620 ORF was generated by introducing four individual point mutations in the ORF obtained for Tb927.8.7630.

**Table S 3 C** - Single nucleotide changes in genes amplified for Tb927.8.7640 from our lab strain of *T. b.*Lister 427 compared to database sequences from TriTrypDB (release 46)

|  | **Database sequences** | | | | | **Our sequences** | | | | | |
| --- | --- | --- | --- | --- | --- | --- | --- | --- | --- | --- | --- |
| **Nucleotide position in Tb927.8.7640** | **Tb927.8.7640** | **Tb427.08.7640** | **AA change** | **Tb427_080082100** | **AA change** | **7640_1** | **AA change** | **7640_2** | **AA change** | **7640_3** | **AA change** |
| 15 | G | G |  | T | K to N | T | K to N | T | K to N | T | K to N |
| 16 | A | A |  | G | T to A | G | T to A | G | T to A | G | T to A |
| 19 | A | A |  | C | K to Q | C | K to Q | C | K to Q | C | K to Q |
| 21 | G | G |  | A | K to Q | A | K to Q | A | K to Q | A | K to Q |
| 22 | T | T |  | C | S to P | C | S to P | C | S to P | C | S to P |
| 24 | G | G |  | A | S to P | A | S to P | A | S to P | A | S to P |
| 27 | A | A |  | C |  | C |  | C |  | C |  |
| 29 | C | C |  | A | T to N | A | T to N | A | T to N | A | T to N |
| 30 | G | G |  | C | T to N | C | T to N | C | T to N | C | T to N |
| 35 | T | T |  | C | V to A | C | V to A | C | V to A | C | V to A |
| 47 | A | A |  | A |  | G | Q to R | A |  | A |  |
| 52 | G | A | D to N | G |  | G |  | G |  | G |  |
| 71 | C | C |  | T | A to V | T | A to V | T | A to V | T | A to V |
| 74 | G | G |  | T | G to V | T | G to V | T | G to V | T | G to V |
| 76 | C | C |  | A | Q to N | A | Q to N | A | Q to N | A | Q to N |
| 78 | A | A |  | C | Q to N | C | Q to N | C | Q to N | C | Q to N |
| 79 | G | G |  | C | A to L | C | A to L | C | A to L | C | A to L |
| 80 | C | C |  | T | A to L | T | A to L | T | A to L | T | A to L |
| 81 | G | G |  | A | A to L | A | A to L | A | A to L | A | A to L |
| 151 | T | T |  | C |  | C |  | C |  | C |  |
| 510 | T | T |  | C |  | T |  | C |  | T |  |
| 751 | C | C |  | C |  | T |  | C |  | T |  |
| 1122 | T | C |  | C |  | C |  | C |  | C |  |
| 1221 | A | T |  | T |  | T |  | T |  | T |  |
| 1353 | C | C |  | T |  | C |  | T |  | C |  |
|  |  |  |  |  |  |  |  |  |  |  |  |
|  |  | **PCR amplicons from genomic DNA** | | | | **3** |  | **2** |  | **1** |  |
|  |  | **Construct in this study** | | | | **7640** |  | **7640’** |  | **-** |  |

The sequence amplified for Tb927.8.7640 (7640) closely resembles the sequence of Tb427_080082100 which differs from the TREU 927 and Lister 427_2010 sequence by 11 amino acids within the first 27 amino acids of the N‑terminus. Because most experiments using these sequences where conducted prior to the publication of the Lister 427_2018 genome, we worked with the sequences closer resembling the 427_2010 genome.

In the main text the cloned ORFs will be referred to as 7610, 7620, 7630 and 7640.
