# Supplementary material for: Nutrient availability regulates proline/alanine transporters in Trypanosoma brucei
Source: J Biol Chem. 2021 Mar 18;296:100566. doi: 10.1016/j.jbc.2021.100566 (PMC8094907; doi:10.1016/j.jbc.2021.100566)
Supplement: Supplementary file 4 — Figures S1 to S13 [file mmc4.pdf]

Nutrient availability regulates proline/alanine transporters in *Trypanosoma brucei*

Alexander C. Haindrich[1], Viona Ernst[1], Arunasalam Naguleswaran[2], Quentin-Florian Oliveres[1], Isabel Roditi[2] and Doris Rentsch[1]*

[1] Institute of Plant Sciences, University of Bern, Bern, Switzerland

[2] Institute of Cell Biology, University of Bern, Bern, Switzerland

*Corresponding author: Doris Rentsch
E-mail: doris.rentsch@ips.unibe.ch

Supporting Figures S 1 – S 13

Figure S 1

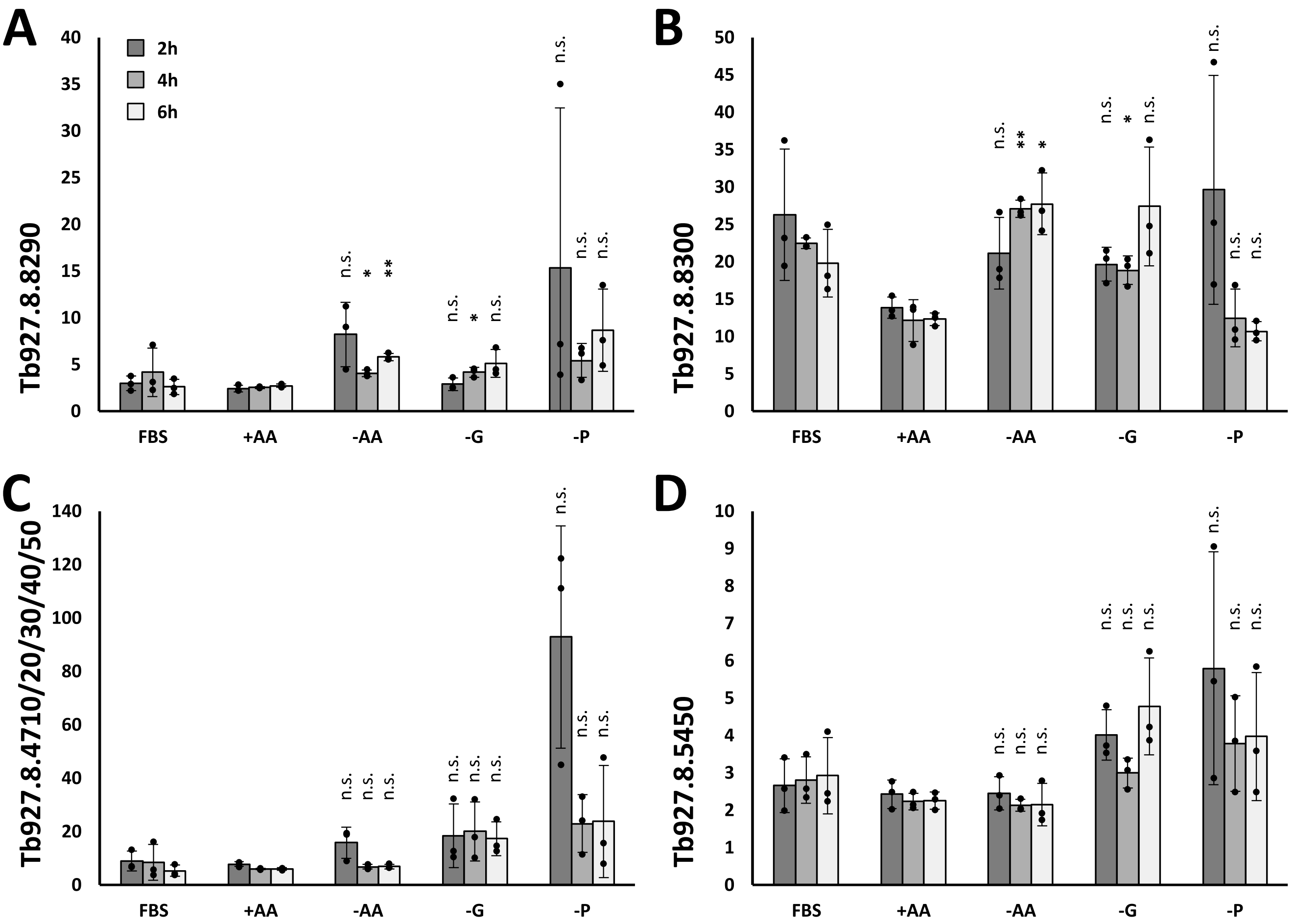

Figure S 1: qRT-PCR analysis of starved PCF. qRT-PCR analysis of amino acid transporter expression of *T. brucei* 29-13 starved for 2h (dark grey), 4h (medium grey) or 6h (light grey) of glucose (G), amino acids (AA) or proline (P). y-Axis shows the expression of A) the ornithine transporter Tb927.8.8290 (AAT10.1, (33)), B) Tb927.8.8300, C) the arginine transporters (Tb927.8.4710/20/30/40/50, AAT5, (31)) and D) the neutral amino acid and eflornithine transporter Tb927.8.5450 (AAT6, (32)), relative to the reference gene TERT. Media composition as described in Table 1 and Table S 1. FBS, commercial SDM79+10%FBS; +AA, starvation medium SDM79S containing amino acids and glucose, but no FBS; -AA, starvation medium SDM79S containing glucose, but no amino acids and no FBS, -G, starvation medium SDM79S containing amino acids, but no glucose and no FBS; -P, starvation medium SDM79S containing amino acids and glucose, but no proline and no FBS. Bars show the average of 3 independent experiments, error bars represent SD, black dots show individual data points. Significance of differential gene expression of cells grown with amino amino acids and glucose was tested against cells starved of amino acids, glucose, or proline, using paired, two-tailed t-tests, for each starvation period individually (*, $p \leq 0.05$; **, $p \leq 0.01$, n.s., not significant).

Figure S 2

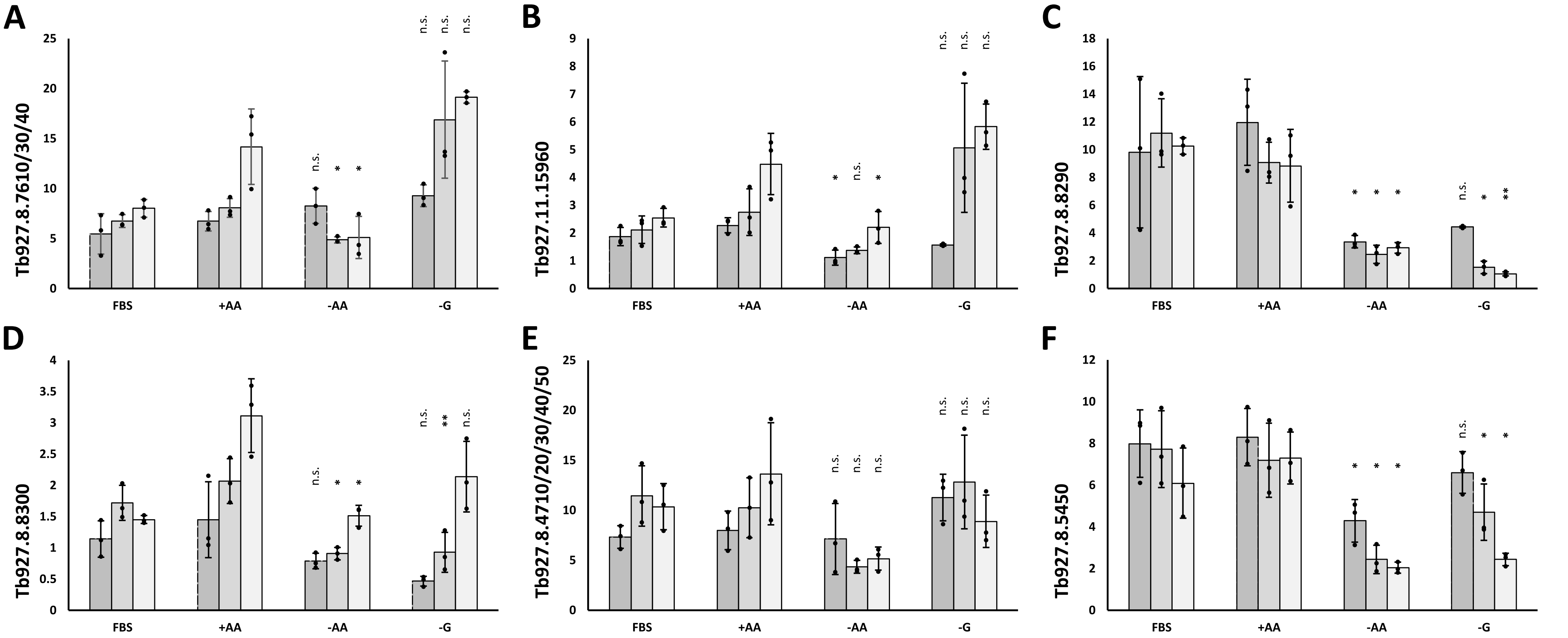

Figure S 2: qRT-PCR analysis of starved BSF. Quantitative RT-PCR analysis for expression levels of amino acid transporters of BSF cells starved for 2h (dark grey), 4h (medium grey) or 6h (light grey) of glucose or amino acids. y-Axis shows the expression of A) Tb927.8.7610/30/40, B) Tb927.11.15960, C) Tb927.8.8290, D) Tb927.8.8300, E) Tb927.8.4710/20/30/40/50 and F) Tb927.8.5450 (AAT6), relative to the reference gene TERT. Media composition as described in Table 1 and Table S 1. Bars show the average of 3 experiments, error bars denote SD, black dots show individual data points. Significance of differential gene expression of cells grown with amino amino acids and glucose was tested against cells starved of amino acids or glucose, using paired, two-tailed t-tests, for each starvation period individually (*, p ≤ 0.05; **, p ≤ 0.01, n.s., not significant).

Figure S 3

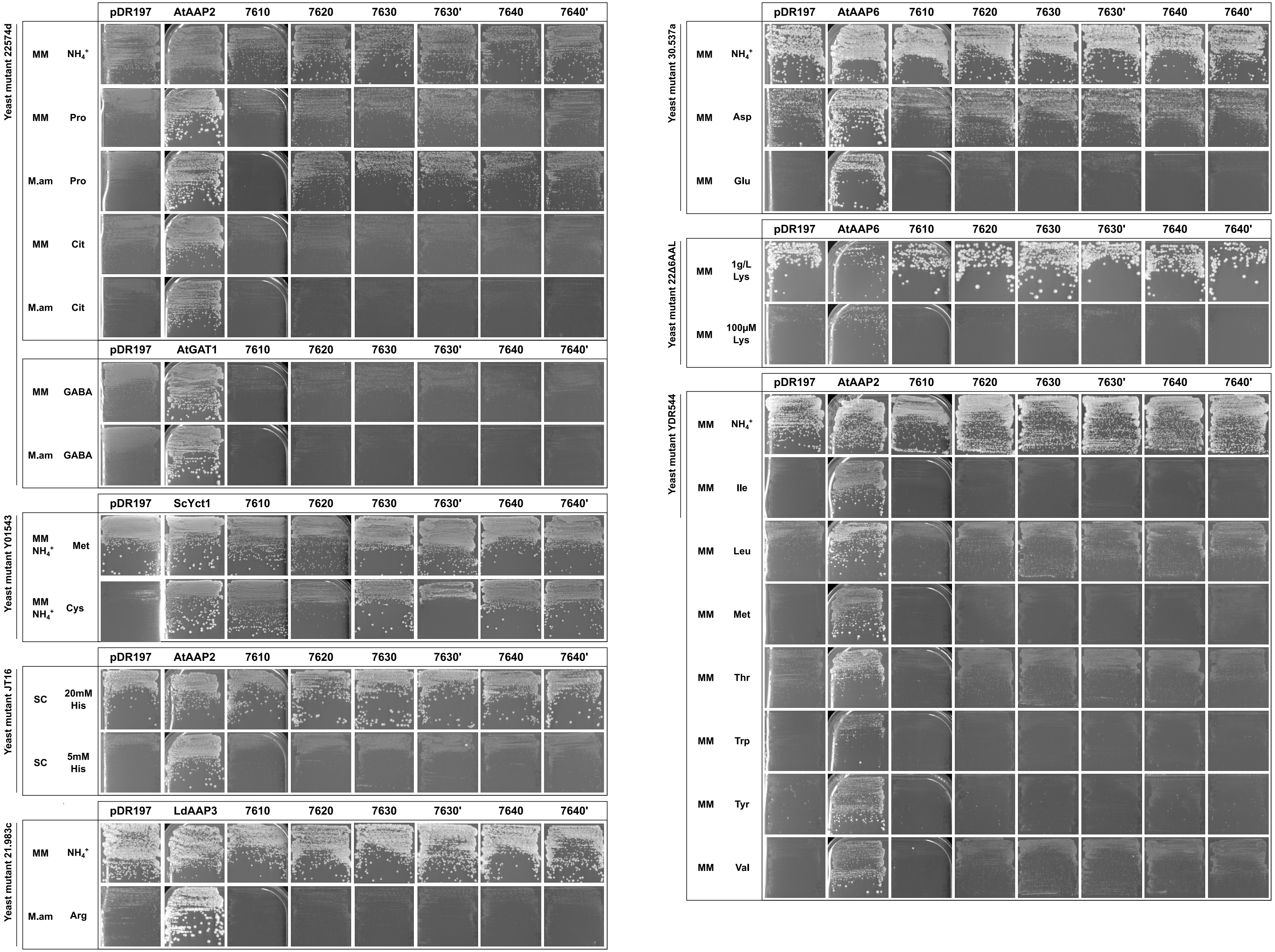

Figure S 3: Complementation of *S. cerevisiae* mutants by AAT7-B members. Growth is shown under non-selective conditions (first row) and using selective concentrations of single amino acids. pDR197 represents the vector control (first column), the second column shows a positive control, i.e. either AtAAP2 (110), AtAAP6 (103), LdAAP3 (105), AtGAT1 (108), or ScYct1 (109). The overexpressed ORFs of the AAT7-B group correspond to the sequences described in **Table S 3**. MM, minimal medium, M.am, minimum buffered (pH 6.1) medium, SC, synthetic complex medium as described in materials and methods.

Figure S 4

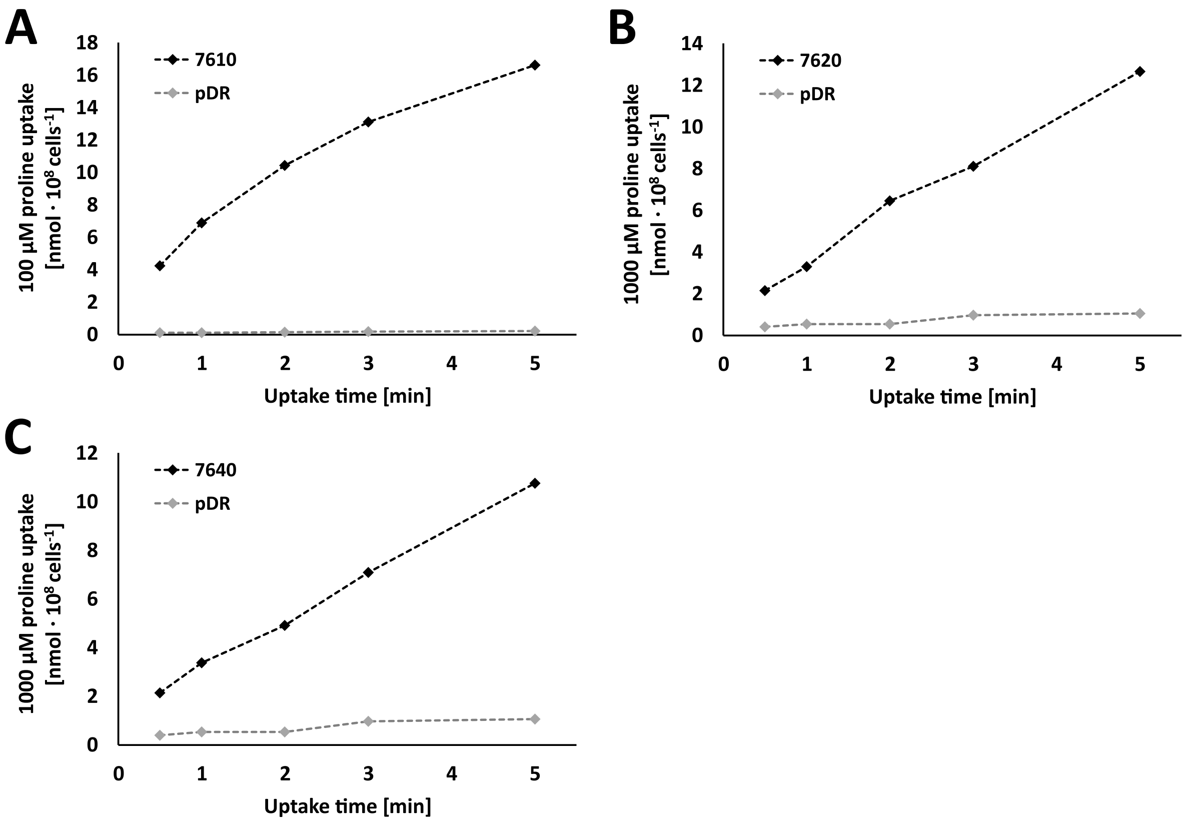

Figure S 4: Time dependent proline uptake of S. cerevisiae strain 22574d expressing 7610, 7620 or 7640. Uptake of 100 µM and 1000 µM L-[3H]-proline was measured for yeast cells expressing the ORFs of A) 7610, B) 7620, or C) 7640, over the time course of 5 min. Uptake measured for the mutant transformed with the vector (background) is also shown. Only data of one exemplary measurement is shown (proline uptake by 7610 was measured at pH 6.5; 7620 and 7640 at pH 7.0).

Figure S 5

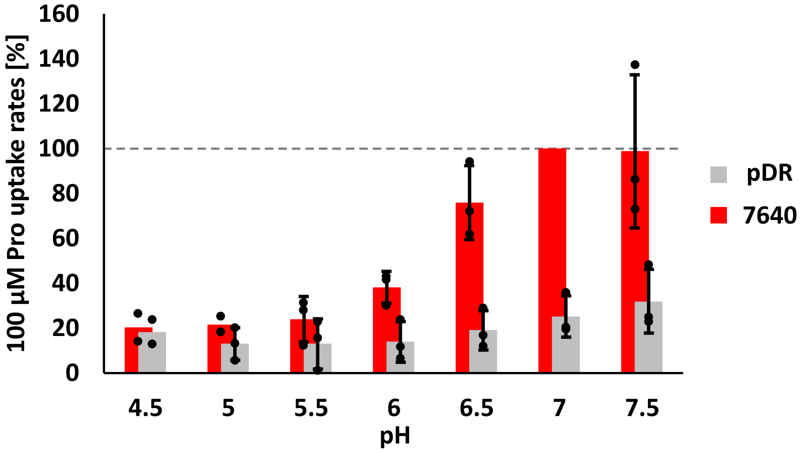

Figure S 5: pH dependent proline uptake rates of S. cerevisiae mutant 22574d expressing 7640. Uptake of 100 µM L-[3H]-proline was measured for yeast cells expression 7640 (red) and the mutant transformed with the vector (pDR, grey). Uptake rates were normalized to the uptake rate of 7640 at pH 7 (=100%). The graph shows the mean of 3 measurements (exception, pH 4.5 and 5 only two measurements, averages based on only 2 measurements show no error bar). Error bars denote SD. Points show the individual measurements.

Figure S 6

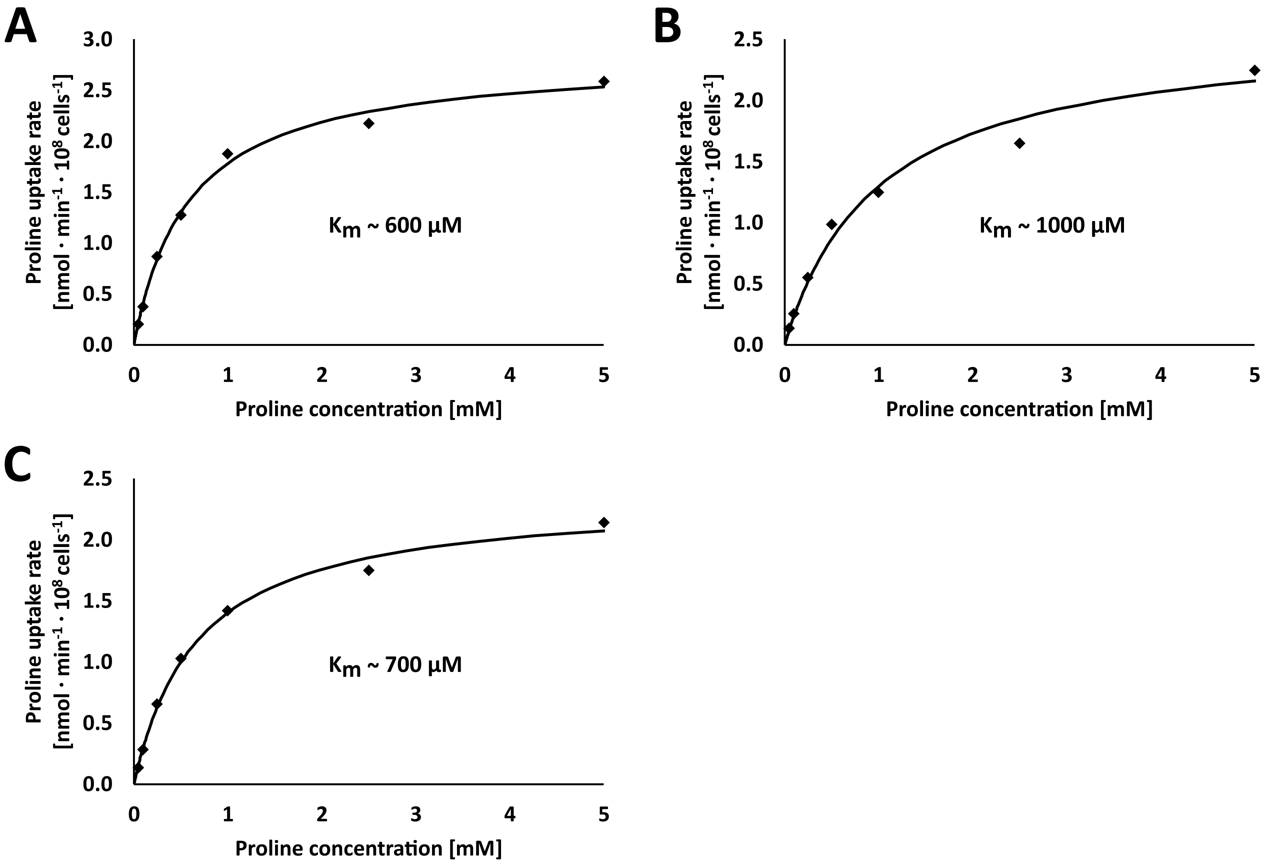

Figure S 6: Estimation of the affinity of 7620, 7630 and 7640 for proline. K_m values were determined for proline using *S. cerevisiae* mutant 22574d expressing A) 7620, B) 7630 or C) 7640. Transport assays were performed at pH 7, using a 5 min time series. Line shows the least square fitted Michaelis Menten curve (n=1).

Figure S 7

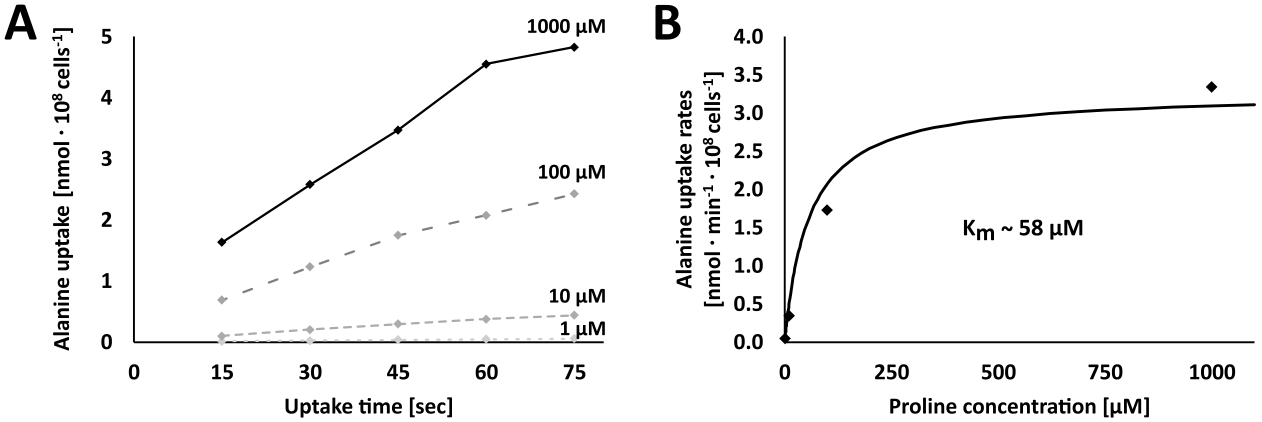

Figure S 7: Uptake of radiolabeled alanine by S. cerevisiae mutant YDR544 expressing 7610. A) Time-dependence of alanine uptake was measured at 4 different alanine concentrations (1 µM, 10 µM, 100 µM and 1000 µM) (pH 7.0, n=1). B) Least square approximation assuming Michaelis Menten kinetic indicates a K_m ~ 58 µM.

Figure S 8

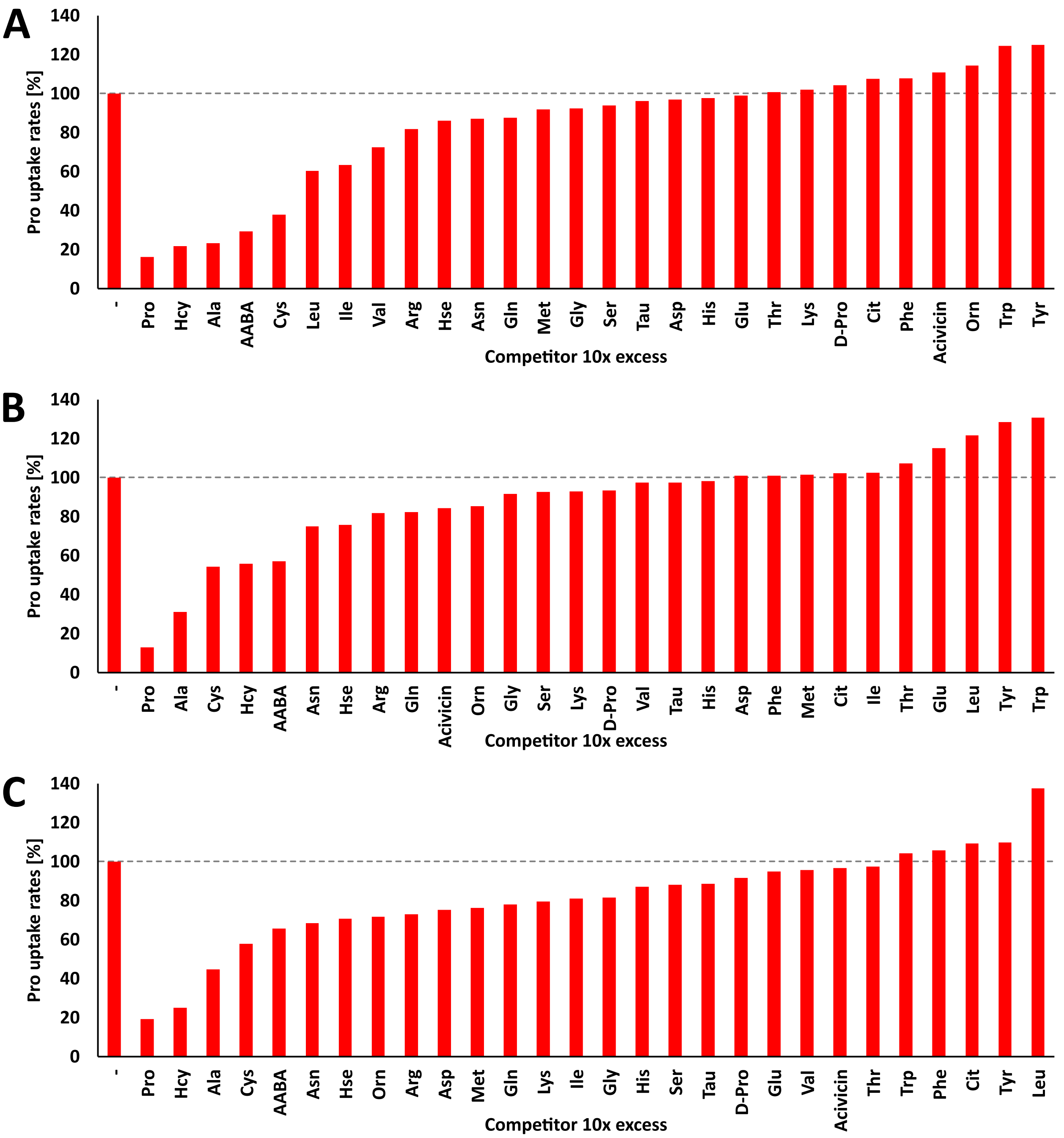

Figure S 8: 7620, 7630 and 7640 preferentially transport proline, but other amino acids are also recognized. Uptake rates of 500 µM L-[^{3}H]-proline were determined in *S. cerevisiae* mutant 22574d expressing A) 7620, B) 7630, or C) 7640, in the presence of different compounds using a 10x excess (5 mM). Uptake rates are shown relative to the control i.e. 500 µM proline, which was set to 100% (100% correspond to 880, 680, 840 pmol min^{-1} 10^8 cells^{-1} for 7620, 7630 and 7640, respectively. Transport assays were performed at pH 7 (n=1). Common L-amino acids are abbreviated with their 3 letter code, other substances are D-proline (D-Pro), α-amino butyric acid (AABA), homoserine (Hse), ornithine (Orn), citrulline (Cit) and homocysteine (Hcy).

Figure S 9

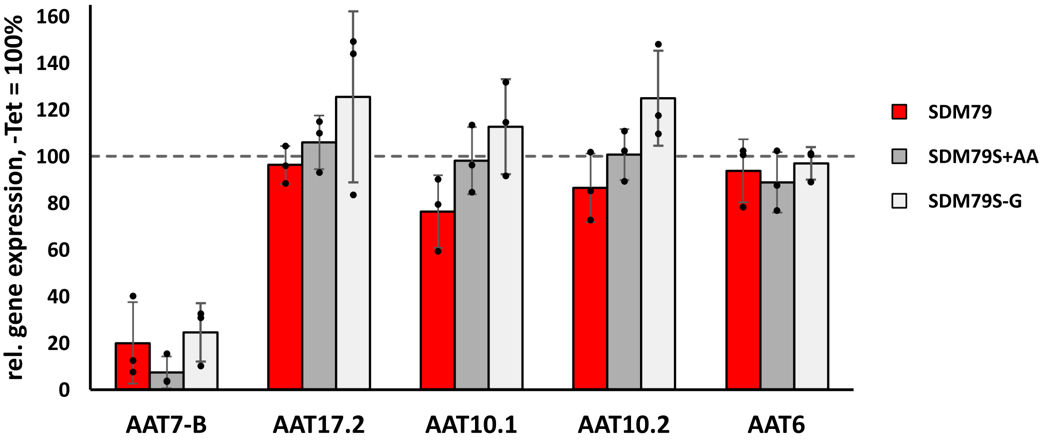

Figure S 9: Expression of amino acid transporters in PCF AAT7-B RNAi cells grown in SDM79, SDM79S+AA, or SDM79S-G. RNA was extracted after 3 days of growth with tetracycline induction (+Tet) or without tetracycline induction (-Tet), and used for qRT-PCR analysis for transcripts of the AAT7-B transporters (AAT7-B, Tb927.8.7610/30/40), Tb927.11.15960 (AAT17.2), Tb927.8.8290 (AAT10.1), Tb927.8.8300 (AAT10.2) and Tb927.8.5450 (AAT6). Bars show the average downregulation relative to non-induced cells of three biological replicates. AN1 was used as reference gene (mean±SD, n=3, dots show measurements from individual replicates).

Figure S 10

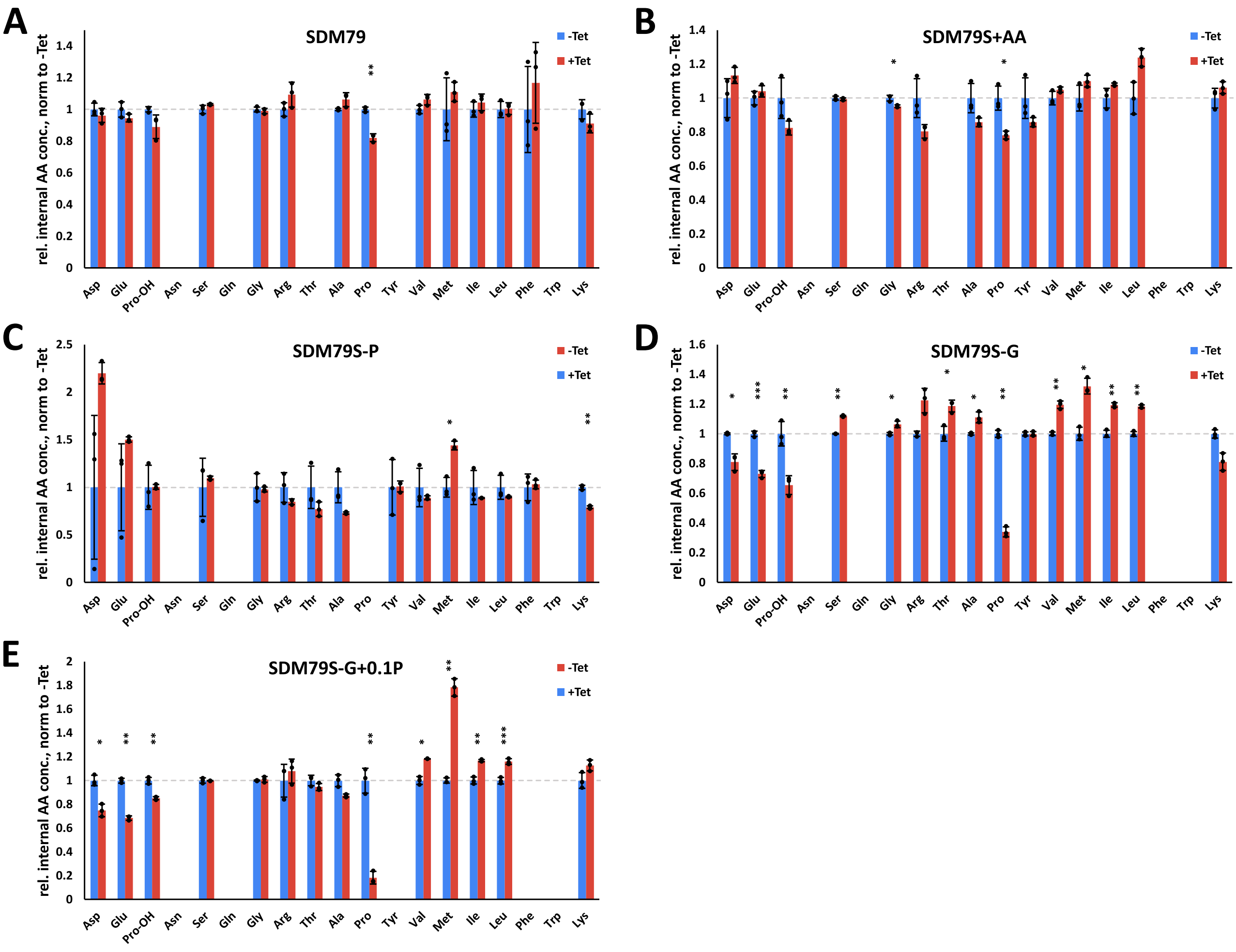

Figure S 10: Amino acid analysis of all detectable intracellular amino acids of PCF AAT7-B RNAi cells grown in SDM79, SDM79S+AA, SDM79S-G, SDM79-P, or SDM79S-G+0.1P, all supplemented with 10% (v/v) FBS. Cells were grown without tetracycline induction (-Tet) or for 3 days with tetracycline induction (+Tet) before amino acids were extracted. Intracellular amino acid concentrations were normalized to the sum of selected amino acids and the average content in non-induced cells was set to 100%. Missing amino acids where either below the detection limit or could not be sufficiently separated from other amino acids or byproducts of the coupling reaction (mean ±SD, n=3, dots represent individual technical replicates, similar results were obtained in an independent biological repeat). Statistical significance was determined using paired, two-tailed t-tests (*, $p \leq 0.05$; **, $p \leq 0.01$; ***, $p \leq 0.001$).

Figure S 11

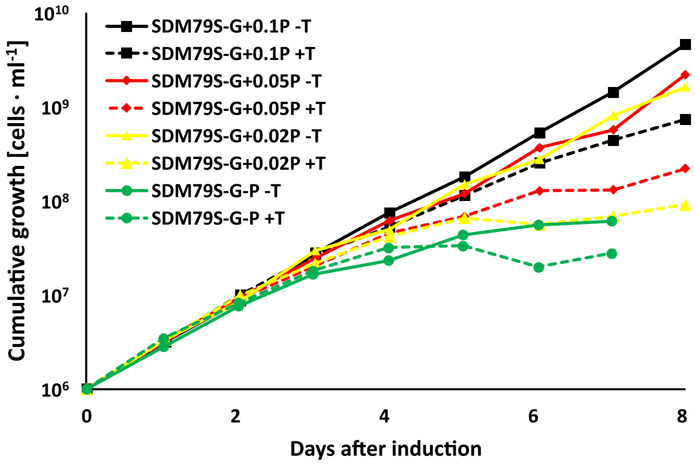

Figure S 11: Minimum proline requirements of PCF T. brucei in the absence of glucose. PCF AAT7-B RNAi cells previously adapted to growth in medium containing no glucose, 5.3 mM proline and supplemented with 10% (v/v) FBS (SDM79S-G) were subsequently cultivated in medium containing no glucose and no proline, but 10% (v/v) FBS (SDM79S-G-P), and also in media containing 10% (SDM79S-G+0.1P, 5% (SDM79S-G+0.05P) or 2% (SDM79S-G+0.02P) of the 5.3 mM proline present in full medium, all containing 10% (v/v) FBS. After the addition of 10% (v/v) FBS to the media, the final proline concentrations are (in descending order) 664 µM, 397 µM, 237 µM or 130 µM. Growth curves were initiated by a 1:1 dilution of cells grown in SDM79S-G with new medium and was continued by dilution of the cells every 24 h to 1x106 cells ml-1 as required, so proline was gradually decreasing. Cells were further grown with (+T, dashed line) or without tetracycline (-T, full line).

Figure S 12

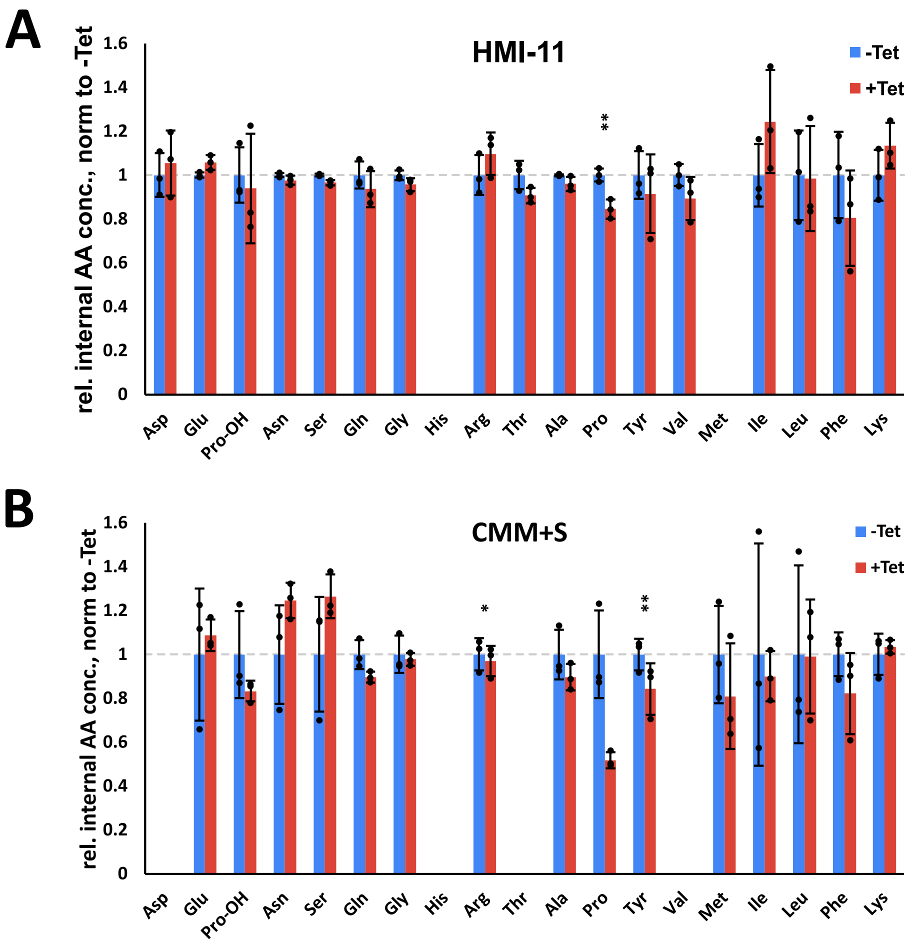

Figure S 12: Amino acid analysis of all detectable intracellular amino acids of BSF AAT7-B RNAi cells grown in media HMI-11 or CMM+S. Cells were grown without tetracycline induction (-Tet) or for 2 days with tetracycline induction (+Tet) before amino acids were extracted. Intracellular amino acid concentrations were normalized to the sum of selected amino acids and the average content in non-induced cells was set to 100%. Missing amino acids were either below the detection limit or could not be sufficiently separated from other amino acids or byproducts of the coupling reaction. (mean±SD, n=3, dots represent individual technical replicates). Statistical significance was determined using paired, two-tailed t-tests (*, p ≤ 0.05; **, p ≤ 0.01).

Figure S 13

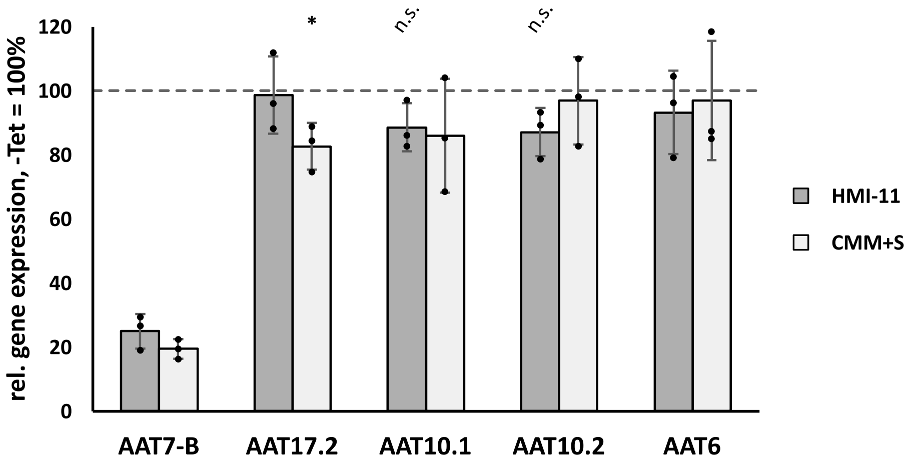

Figure S 13: qRT-PCR analysis of amino acid transporter transcript levels in BSF AAT7-B RNAi cells grown in HMI-11, or CMM+S. RNA was extracted after 2 days of growth with tetracycline induction (+Tet) or without tetracycline induction (-Tet), and used for analysis of the transcripts of the AAT7-B transporters (AAT7-B, Tb927.8.7610/30/40), Tb927.11.15960 (AAT17.2), Tb927.8.8290 (AAT10.1), Tb927.8.8300 (AAT10.2), and Tb927.8.5450 (AAT6). Bars show the average downregulation relative to non-induced cells of three biological replicates. TERT was used as reference genes for the quantification of AAT7-B, other genes were quantified using AN1 as reference gene (mean±SD, n=3, dots show measurements from individual replicates). Statistical significance was determined using paired, two-tailed t-tests (*, $p \leq 0.05$; n.s., not significant).
